# Microbes little helpers and suppliers for therapeutic asthma approaches

**DOI:** 10.1186/s12931-023-02660-7

**Published:** 2024-01-13

**Authors:** Sebastian Reuter, Jonas Raspe, Christian Taube

**Affiliations:** Department of Pulmonary Medicine, University Hospital Essen-Ruhrlandklinik, Tüschener Weg 40, 45239 Essen, Germany

**Keywords:** Asthma, Microbiome, Gut–lung axis, Inflammation, Therapy, Treatment

## Abstract

Bronchial asthma is a prevalent and increasingly chronic inflammatory lung disease affecting over 300 million people globally. Initially considered an allergic disorder driven by mast cells and eosinophils, asthma is now recognized as a complex syndrome with various clinical phenotypes and immunological endotypes. These encompass type 2 inflammatory endotypes characterized by interleukin (IL)-4, IL-5, and IL-13 dominance, alongside others featuring mixed or non-eosinophilic inflammation. Therapeutic success varies significantly based on asthma phenotypes, with inhaled corticosteroids and beta-2 agonists effective for milder forms, but limited in severe cases. Novel antibody-based therapies have shown promise, primarily for severe allergic and type 2-high asthma. To address this gap, novel treatment strategies are essential for better control of asthma pathology, prevention, and exacerbation reduction. One promising approach involves stimulating endogenous anti-inflammatory responses through regulatory T cells (Tregs). Tregs play a vital role in maintaining immune homeostasis, preventing autoimmunity, and mitigating excessive inflammation after pathogenic encounters. Tregs have demonstrated their ability to control both type 2-high and type 2-low inflammation in murine models and dampen human cell-dependent allergic airway inflammation. Furthermore, microbes, typically associated with disease development, have shown immune-dampening properties that could be harnessed for therapeutic benefits. Both commensal microbiota and pathogenic microbes have demonstrated potential in bacterial-host interactions for therapeutic purposes. This review explores microbe-associated approaches as potential treatments for inflammatory diseases, shedding light on current and future therapeutics.

## Background

Bronchial asthma is a chronic inflammatory lung disease that affects more than 300 million people worldwide and is increasing in prevalence [1]. First described as an allergic disorder of the lower airways driven by mast cells and eosinophils, asthma is now understood to be a heterogeneous syndrome with different clinical phenotypes, pathogenesis and underlying immunological endotypes. These range from type 2 inflammatory endotypes that are dominated by interleukin (IL)-4, IL-5 and IL-13, to other types where no eosinophilic inflammation is detected in the airways or mixed inflammation with type 1 and type 17 cytokines.

The complexity of the different pathophysiological mechanisms underlying asthma is also mirrored by the therapeutic success of different therapies in the varying phenotypes. Individuals with milder forms asthma benefit from treatment with inhaled corticosteroids and beta-2 agonists, while those with more severe disease often show poor or no response to these conventional therapies. In recent years, novel antibody-based approaches have been developed for certain phenotypes of asthma. The main application for these treatments to date has been severe allergic asthma and severe asthma and type 2 high inflammation or eosinophilic inflammation. In individuals with the relevant asthma phenotype and severe disease, these novel therapeutics help to reduce exacerbation rates and improve quality of life. In 2022, an anti-thymic stromal lymphopoietin (TSLP) antibody was approved for use in Europe for the treatment of severe asthma in patients with no phenotype or biomarker limitations [2] This agent, tezepelumab, has shown some effectiveness in individuals with type 2-low asthma, however, the observed effects are less convincing compared to the effectiveness in type 2-high patients. There is still a therapeutic gap for patients with severe type 2-low asthma.

Based on available data, there is a need for novel treatment strategies that provide better control of the pathology driving inflammatory processes to prevent asthma development or disease exacerbations. In particular, it would be interesting to determine the long-lasting effects of therapeutic interventions that stimulate endogenous anti-inflammatory responses by inducing and activating regulatory T cells (Tregs). Together with T helper cells Tregs belong to the fraction of CD4^+^ T cells. They can differentiate in the thymus or only in the periphery, so that one commonly differentiates between naturally occurring thymus-derived tTregs and induced pTregs. Unlike effector T cells, Tregs are responsible for maintaining immune homeostasis, preventing autoimmunity, and eliminating/preventing excessive inflammatory responses after contact with pathogens or pollutants [3]. In murine models, Treg cells can control type 2-high and type 2-low inflammation [4], and dampen human cell-dependent allergic airway inflammation in the lung [5]. This suppression is mediated via the release of anti-inflammatory cytokines (such as IL-10, transforming growth factor [TGF]-β, and IL-35) or via cell–cell contacts. In addition, Tregs can also indirectly throttle immune cell activity via interaction with dendritic cells (DC) and degradation of metabolically essential products (e.g., adenosine triphosphate or tryptophan).

Surprisingly, although normally associated with the development, progression or exacerbation of diseases, microbes have emerged as potential beneficial tools that have immune dampening properties. Both commensal bacteria forming the microbiome and microbes normally seen as pathogens have shown bacterial-host interactions with potential therapeutic suitability. This review will highlight several microbe-associated approaches representing current or future potential therapeutics for the treatment of inflammatory diseases.

## The other face of bacteria: symbiotic or commensal roommates

The role of microbes as hostile intruders that are responsible for the development of infectious diseases that could have life-threatening consequences is well known. Infections are associated with asthma and, in particular, exposure to certain viruses is associated with the development of asthma and acute disease exacerbations [6]. The interactions between microbes and humans are complex, ranging from pathological destructiveness to indifferent coexistence and symbiotic cohabitation. Based on these different forms of interaction, several hypotheses have been developed stating that beneficial interactions between microbes and host can prevent diseases, while the absence of microbial species, due to changes in lifestyle (e.g. excessive hygiene or use of antibiotics) can be responsible for disease development.

In his “hygiene hypothesis”, Strachan was one of the first to postulate that infections in early childhood and improved hygiene standards in developed countries are responsible for an increased risk of developing allergies [7]. Further studies and developments in gnotobiotic animal research showed a protective role of various environmental bacteria and commensal bacteria, forming the indigenous microbiota, on the development of atopy.

Culturing of anaerobes and new high-throughput methodology such as matrix-assisted laser desorption/ionization-time of flight (MALDI-TOF), 16S RNA sequencing, phylogenetic microarrays or taxon targeted qPCR, have shown the diversity of bacterial species colonizing environmental-exposed organs including the gut and skin, but also the lung. Furthermore, methods like metabolomics, proteomics, transcriptomics, metagenomics or single cell sequencing have provided additional insights into the physiological function of our body mates.

Based on data obtained using these techniques, it was found that the microbiota in one healthy human subject consists of approximately 30 trillion microbes, with the largest proportion being bacteria, followed by viruses (bacteriophages and human viruses), and yeasts [8]. In healthy individuals, the composition of the microbiome is quite stable but differs between organs or even within sections of the same organ. In the gastrointestinal tract, the functions of the microbiome are already quite well described. Several studies have shown an impact of the microbiome on the development of immunity [9], host defense [10], metabolic supply [11], fat storage [12], synthesis of vitamins [13] and even an association with behavior [14]. Microbial products have many functions: defense systems can protect from pathogens, while ligands and nutrients can perform intra-microbial communication, and inter-kingdom communication between microbe and host. Signals can act as short-distance messengers but are also able communicate with distant organs [15]. Pathogen-associated molecular patterns (PAMPS), and metabolic products like indole-3-aldehyde (a ligand to the aryl hydrocarbon receptor [AHR]) or short fatty acids modulate host immune responses and therefore mediate both pro- and anti-inflammatory responses. These are associated with the development and maintenance of healthy immune homeostasis. Interestingly, changes in the microbiome-host relationship are associated with several diseases in different organs, such as autism, stress or stroke (brain), asthma (lung), atopic dermatitis (skin), inflammation and obesity (adipose tissue), and others such as type 3 diabetes, systemic lupus erythematosus or atherosclerosis.

There are different reasons for these associations. Pathophysiological changes due to disease, exogenous stressors, medication [e.g. antibiotics] and changes in diet can all affect the composition of the microbiome and drive host interaction malfunction. The identification of beneficial microbial strains and their restoration to regain health-promoting function could be a therapeutic approach for various diseases. Different strategies helping to restore, complement or replace ineffective microbiomes are in development. Targeted treatments with antibiotics or bacteriophages are thought to destroy pathogenic bacterial species. Mills et al. showed that phages naturally shape host-associated bacterial populations [16]. With new gene editing methods, it will be possible to design specific phages to target specific unsuitable bacterial species at an individual patient level [17]. This will terminate pathogenic processes and create space for the expansion of beneficial species. To further support repopulation with desirable species, targeted transfer of single beneficial or genetically modified species (probiotics), designed communities, or multispecies or whole microbiome applications by fecal transplantations (FMT) can be performed. For example, Sheng et al. showed that FMT is a beneficial treatment option in children with infantile allergic colitis refractory to standard therapeutics [18].

However, it should not go unmentioned that these methods still present us with a number of challenges. With FMT in particular, it is clear that rigorous screening of donors and recipients is important to ensure the success of a healthy microbiome transfer and to avoid potential side effects such as the transfer of antibiotic-resistant bacteria or the induction of sepsis.

Due to the increasing simplicity of methods for genetic modification of microorganisms, bacteria of the microbiota themselves are being considered as therapeutic tools. This is especially the case for bacteria such as *Escherichia coli Nissle* [19], *Lactobacillus* or *Lactococcus* [20], which tend to induce anti-inflammatory immune responses in the host and are not capable of long-term colonization. Designed bacteria are capable of supporting the formation of a healthy microbiota and producing compounds that support beneficial metabolic pathways, or destroy or prevent pathogenic processes [19]. In addition to the supplementation of bacteria, diets with selected nutrients and the use of prebiotics or synbiotics can enhance the development of metabolites with beneficial effects.

Taken together, maintenance of a healthy microbiota and support for the development of desirable metabolites provide a “natural” therapeutic tool to prevent, treat or at least support the treatment of a wide range of diseases. New methods allowing personalized examinations to provide detailed characterization of the host microbiota will help to optimize individual treatment strategies.

## The lung and the microbiome

The long-held dogma that the lungs are a sterile organ meant that it was not considered to be an area that contained a microbiome. However, almost a decade after initiation of the human microbiome project in 2007 [21], the first studies revealed a microbial microcosm in the healthy lung [22, 23]. Still, limited access to sample material from healthy lungs and concerns about contaminations during the sample collection process slowed the research. Today, with the emergency of methods like 16S rRNA analysis, this view has changed. A healthy lung microbiota, which is formed from different bacteria including members of the *Protobacteria*, *Firmicutes*, *Actionbacteria* and Bacteroidetes phyla, has been identified and is now accepted [24]. In healthy people, the lung microbiome shares many similarities with the upper airway microbiome; probably caused by aspirations of oropharyngeal fluids [25]. Furthermore, shifts in the composition of the bacterial communities of the lung microbiota is associated with different lung diseases. Specific changes in the lung microbiome have been found in individuals with asthma [26–31], while different changes have been associated with chronic obstructive pulmonary disease (COPD) [26, 32] or cystic fibrosis [33].

An important role for the microbiome has been identified for susceptibility to asthma. Data from germ-free or antibiotic-treated murine models have shown a strong relationship between the microbiome and the development of asthma [34–36]. In this setting there appears to be a “time window of opportunity” during pregnancy and especially in the first years of life that seems to be important for the development of a healthy protective microbiome.

The antibiotic animal models served to emphasize the role of the microbiome as a beneficial early childhood factor that can reduce the risk of developing diseases later in life. Under no circumstances should they cast doubt on the usefulness of antibiotics for the treatment of potentially life-threatening infectious diseases, but they should encourage the sensible use of these drugs. In this context, it is also worth mentioning that that the timing of antibiotic treatment, such as azithromycin, plays an important role. While mouse model confirm that early life treatment with azithromycin increased the susceptility to develop allergic asthma in later life, epidemiological studies on the effect of antibiotics on the course of lung disease in older children are controversial [37]. On the one hand, no positive effects on recurrent wheezing after RSV bronchiolitis could be observed with parallel treatment with the antibiotic [38, 39]; on the other hand, early treatment in children who frequently suffer from severe episodes of lower respiratory tract illness (LRTI) led to a milder course of the disease [40]. The exact role of the microbiome in asthma is not yet fully understood. What is clear is that there are differences between healthy people and those with asthma. Whether there are also differences in the microbiome depending on asthma severity remains controversial. Some think that the microbiome does not differ between asthma phenotypes [41, 42], while other studies show that the microbiome of individuals with severe asthma is associated with corticosteroid insensitivity and eosinophils [43, 44]. Nevertheless, there are several disease-driving functions that are likely to be affected by the composition of the lung microbiome. For example, interactions with the immune system can affect the inflammatory profile or corticosteroid responsiveness and so dramatically influence the course of the disease.

**Fig. 1:**
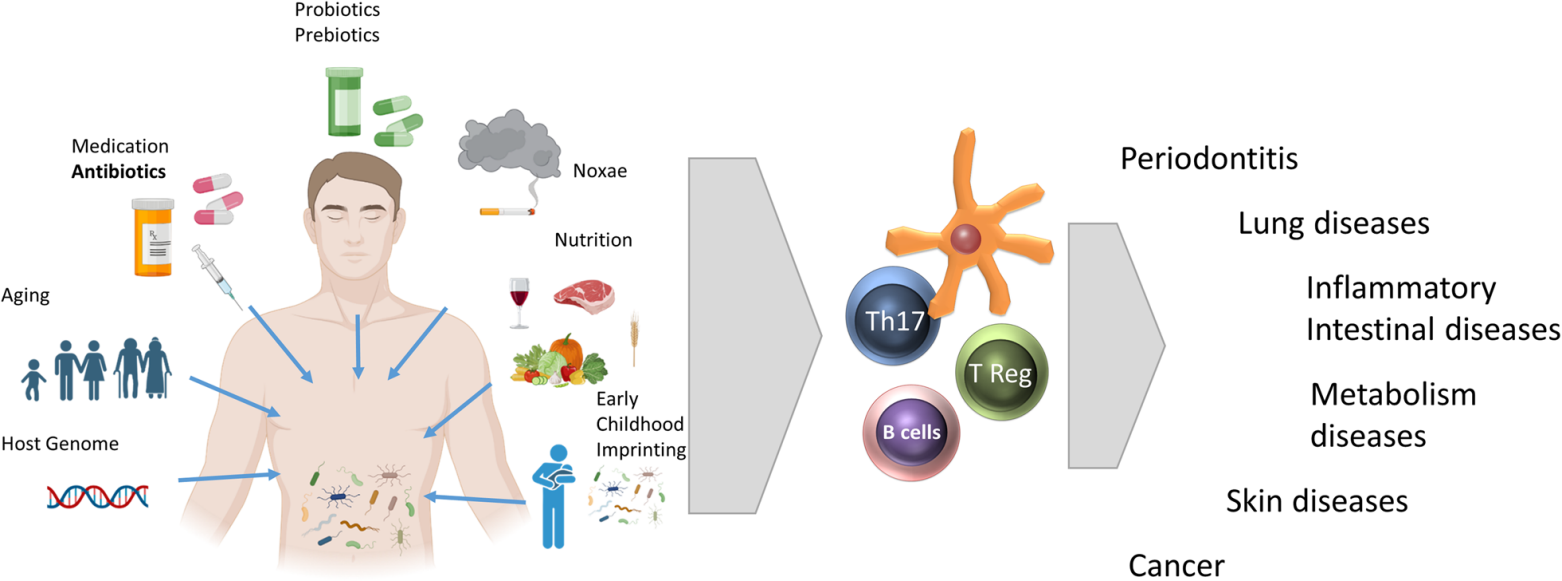
Factors influencing the composition of the microbiome. These factors include host characteristics, such as genetic factors or age, the use of drugs (antibiotics) or pre- and probiotics, environmental factors, nutrition or early childhood factors (birth and feeding mode). Changes often have consequences on the interaction of the microbiome with the immune system and can affect the development and progression of diseases

Positive manipulation of the lung microbiome could have beneficial therapeutic effects. Although data concerning a direct therapeutic manipulation of the human lung microbiome are scarce, we can expect that every environmental manipulation supporting the development of a “healthy” microbiome will reduce susceptibility to the development of atopy. It has already been shown for other organs, especially the gastrointestinal tract, that different exogenous factors such as supplementation of omega-3 fatty acids or vitamin D during pregnancy [45], natural delivery [46, 47] breastfeeding [48], and the avoidance of maternal antibiotics have been associated with a reduction in the risk of asthma development by shaping a healthy microbiome (Fig. 1).

## Microbiome-immune system interaction: a relationship with an educational mission

The innate and adaptive immune systems represent an endogenous task force responsible for defense against exogenous, potentially harmful, intruders and the maintenance of homeostasis, respectively. Forming a complex network, immune cells are present in, or can be recruited within minutes to, all tissues. A sophisticated control and balancing of immune cells and their mediators is essential to provide appropriate and effective responses against pathogens and harmful substances while preventing overwhelming potentially destructive inflammatory reactions or misguided responses against innocuous stimuli. Malfunction in this finely-tuned control of immunity is responsible for the development, progression and exacerbation of various diseases.

Co-evolved towards mutualism, interactions between the immune system and the microbiome play a central role in the development and induction of proper immune functions. In the first years of life, the microbiome plays a central role in the maturation of a variable, unorganized infantile immune system to an effective, organized adult set-up. Studies in germ-free animals showed that an abundant microbiota led to defects in gastrointestinal tract lymphoid cells, monocytes, and the production of and sensitivity to antibodies [49]. This shaping of immunity in the early life “window of opportunity” seems to mediate long-lasting beneficial effects on homeostasis and adequate host defense. In particular, interplay between structural cells (such as epithelial cells), dendritic cells and the microbes is thought to play a key role in microbiome-mediated immune regulation. Pattern recognition receptors on both endogenous cell types are able to sense bacterial structures and mediate both pro- and anti-inflammatory signals. Epithelial cells of the intestine can express Toll-like receptors (TLR; -1,-2,-3,-5,-9) and nucleotide oligomerization domain 2 (NOD2). They can interact directly with immune cells by the expression of chemokines, cytokines and major histocompatibility complex (MHC) I and MHCII. Moreover, they are also able to directly modulate the composition of the gut microbiota via expression of anti-microbial peptides [50].

Epithelial cells are in close proximity to intraepithelial lymphocytes, which can mediate both structural protection and inflammation. Right beside these first-line defenders lays the lamina propria, which is populated with T and B lymphocytes and DC. These cells are able to exert both pro- and anti-inflammatory responses. Anti-inflammatory responses are mediated by Tregs induced especially by CD103^+^ DC, whereas CD103^−^ DC are associated with inflammation and the activation of IL-4, interferon (IFN)-γ, IL-22 or IL-17 secreting effector T cells. While it is most likely that immune regulation is mediated by the entire microbiome, most of the findings relating to microbiome-host interactions come from single bacteria species studies. Here, impact on induction of anti-inflammatory Tregs, activation of NK cells and Th17/22 cells, or development of IgA-producing B cells could be observed.

Their ability to sense a plurality of endo- and exogeneous danger signals, to uptake, process and present antigens via MHCI and MHCII, and to produce chemokines and cytokines make DC a professional antigen-presenting cell and a central element in the regulation of adaptive immunity. The type and strength of activation signal regulates the maturation state and determines the nature of the immune response; tolerance or sensitization. DC play an essential role in the induction of T cell and B cell responses. They are able to directly or indirectly modulate T cell subtypes and class-switch of B cells via expression of immune activating but also inhibitory motifs and the release of different mediators.

As a result, it is not surprisingly that DC play a central role in mediating the communication between microbiome and adaptive immunity. DC activation and subsequently induced T cell response seem to be differentially regulated depending on type of commensal bacteria [51]. Interestingly, application of the mixture IRT5 containing microbiome-associated bacterial species is able to induce DC with a tolerogenic phenotype. These DC are able to induce regulatory T cells and have beneficial effects in different diseases like inflammatory bowel disease [52], atopic dermatitis [53], rheumatoid arthritis [54] or myasthenia gravis [55].

One approach to enhancing immune-suppressing properties is to eliminate pro-inflammatory bacterial compounds. For example, lipoteichoic acid (LTA) is major membrane component of gram-positive bacteria and a well-known antagonist of TLR-2. Several studies showed that Lactobacillus species deficient in LTA can mediate anti-inflammatory responses and induce regulatory DC [56, 57]. The effects of bacterial compounds seem to differ between bacterial species, reports on the functional role of LTA are controversial, and this seems to depend on both strain and immunological milieu [58–60]. Both pro- and anti-inflammatory effects are also described for other compounds like peptidoglycan (PGN). Fernandez et al. reported that PGN derived from *Lactobacillus salivarius Ls33* was capable to induce anti-inflammatory DC, while *L. acidophilus* failed to mediate protection [61].

This is also the case for *Bifidobacterium adolescentis* strains. Depending on the strain, differences in DC-specific IL-6, TNF-α, IL-10 induction have been seen, with consequences for the ratio of developing Th17 cells and Tregs. Jeon et al. further analyzed the effectiveness of different intestinal bacteria capable of promoting the induction of regulatory T cells. In their studies *Bifidobacteria breve* but not *Lactobacillus casei* were able to induce Tregs by a DC IL-10- and IL-27-dependent mechanism [62].

Taken together, these data demonstrate that the interaction between the microbiome and DC is complex. Depending on the type of bacteria, differences between species of the same genus and differences in comparable molecules between species, both inflammatory and tolerogenic DC phenotypes can be induced. These promote antigen-specific responses, but also modulate “bystander” immune responses, and therefore influence the pathology of diseases. For example, in a murine tumor model, the elimination of gram-positive bacteria led to a more effective DC-dependent anti-tumor response following radiotherapy [63].

Originating from DC, activation and differentiation of T cells is another important step that determines adaptive immunity. Activation of naïve CD8^+^ cytotoxic T cells and differentiation of CD4^+^ T helper cells is important for the type and strength, and the abrogation, of immunological responses. Effector T helper cells can differentiate in a variety of subclasses with specific cytokine profiles and are so capable in modulate B cell and innate immune responses. However, in addition to these pro-inflammatory T cells, Treg can also be activated by DC, which can exert immunosuppressive effects by means of cytokines or by direct cell contact.

Commensal bacteria of the gastrointestinal tract control T cell homeostasis by regulating balance between inflammation-inducing Th17 cells and inflammation-suppressing IL-10-producing Tregs.

Bacterial strains belonging to the microbiome, like *Bacteroides fragilis* or several *Clostridium* strains, have been positively associated with the induction of Tregs. Interestingly, *Clostridium* strains, devoid of toxins or virulence factors, are strong inducers of Tregs. The generation of Tregs seems to be dependent on microbial products. Metabolites like Bacteroides-derived polysaccharide A [64] and short chain fatty acids (SCFA) [65, 66] were identified as major drivers for immune protection. In particular, SCFA metabolites butyrate and propionate, but not acetate, have Treg inducing properties [67]. SCFA exert their immune regulatory function by inhibiting histone deacetylases and are able to induce both pro- and anti-inflammatory effects [68].

These modulations are thought to protect degradation of FoxP3 proteins and to induce their expression, thereby mediating both the induction and stability of Tregs [67]. Moreover, SCFA appear to be capable of inducing CD103 + tolerogenic DC [69] and IL-10-producing B cells (Bregs) [70]. The immune dampening effects are thought to mediate tolerance against microbial antigens, thus supporting the cohabitation between host and microbial guest. As a positive side effect, SCFA also mediate immune suppression to food allergens. Modulation SCFA metabolism, for example by special SCFA-containing diets, is thought to be a therapeutic approach for food allergy [71, 72]. Interestingly, SCFA did not only protect from food but also from other not gut related allergies (see next section).

Similar to SCFA, the zwitterionic capsular polysaccharide A (PSA), derived from *B. fragilis,* demonstrated T cell-dependent regulatory properties. Oral application of PSA induced IL-10 producing CD4^+^FoxP3^−^ T cells that attenuated inflammatory responses in a murine asthma model [73].

Treg and IL-10-positive B cell inducing capacities have also been described as necessary to protect against inflammatory bowel disease [64, 73]. In humans, PSA support Treg stability [74]. Interestingly, PSA-induced Tregs suppressed Th17 cells, supporting the idea that *B. fragilis* induces host Tregs to prevent counter measure and promote its colonization. Detailed analysis of T cells showed that PSA is able to modulate both inflammatory cytokine profiles with induction of regulatory surface marker profiles on T cells [75]. Again, these observations demonstrate the ability of a microbial compound to induce context-dependent both pro- and anti-inflammatory responses.

While induction of Tregs and IL-10 is associated with immune suppression, IL-17 is associated with anti-bacterial inflammatory responses and often accompanied by neutrophilia. Several bacterial strains of the microbiota demonstrate IL-17-inducing properties. IL-17 seems to be necessary to maintain immune homeostasis and promote appropriate communication with commensal bacteria, thereby preventing induction of inflammatory responses. Altered composition or dysbiosis of gut microbiota and infectious contact with pathogens can change the role of IL-17 towards a pro-inflammatory disease-driving molecule [76].

Microbiota-associated bacteria like cytophaga-flavobacter-bacter-oidetes (CFB) [77] or segmented filamentous bacteria (SFB) [78] seem to be two central IL-17 triggers. Changes in the composition of these bacteria affect the Tregs/Th17 ratio [77]. Imbalances in this complicated relationship are responsible for the induction and severity of several diseases, including COPD [79], systemic sclerosis [80, 81]; thrombocytopenia, GVHD [82, 83], and asthma [84]. Shifting the equilibrium to immune suppression by supporting the induction of Tregs or preventing the development of IL-17-producing cells will have beneficial effects for numerous diseases.

## The far-reaching arm of the gut

Microbes of the gastrointestinal tract microbiota exist in close proximity to the host but fail to cross epithelial barriers and reach the inside of the body. Breach of this compartmentalization can induce massive inflammatory responses that often have drastic consequences for the host. A reduction of the epithelial integrity can result in a “leaky gut”. Bacteria can now reach sterile tissues and body regions, and activate innate and adaptive immunity. Depending on the extent of the leakage, this change in host and microbiome communication could also contribute to the development of chronic systemic diseases (e.g. stress-related psychiatric disorders like depression [85], heart failure [86]) or acute life-threatening conditions like sepsis.

Transfer of microbial compounds or metabolic products across the epithelial border affects local organs and systemic processes in a beneficial way. One of the main pathways of long-reaching immune modulation within the gut–lung axis is the mesenteric lymphatic system. Through this system, metabolites can translocate across the intestinal barrier and modulate immune responses [87]. While immune system-triggering factors like LPS, flagellin, peptidoglycan and other PAMPS are generally not translocated across the epithelial barrier, bacterial metabolites are capable of entering the body. Here, they are mainly associated with beneficial, but also sometimes negative, effects. SCFA, for example, is involved in energy metabolism [88], to modulate pancreatic function and insulin release, and regulate appetite [89] and glucogenesis [90]. Lactobacilli-derived indole-3-aldehyde or bile-acids are involved in mucosal homeostasis [91]. Bile acids produced by the liver and modified by the microbiota can act as secondary hormones and modulate responses in adipose tissue, kidneys, heart or the enterohepatic circulation [92].

Precise information concerning the mechanisms describing the interplay between the microbiome and other organs have been reviewed elsewhere (e.g. cardiovascular system [93], liver [94], adipose tissue [95] and the brain [96]. Communication between several different tissues, such as the cardiovascular system, liver, adipose tissue, brain and lung have been described. These observations led to the development of terms like the “gut-brain” or “gut–lung axis” to refer to the complex relationship between the gut microbiome and its impact on disease-promoting or preventing processes in peripheral organs.

Next we will focus on cross-talk between the lung and the gut microbiome. Observations that gut dysbiosis is associated with asthma development in children [97, 98] contributed to the hypothesis that bacteria in the gut have a beneficial effect in preventing inappropriate immune responses towards harmless antigens in later life. Children with a reduced abundance of bacterial genera like *Lachnospira, Veillonella, Faecalibacterium*, and *Rothia* had an increased susceptibility to develop asthma in later life [99]. A humanized microbiota mouse model confirmed these observations and showed that the early time window after birth is critical for the development of an atopy preventing microbiota [100]. In addition, early-life colonization with species like *Clostridium difficile* [101] or *Lactobacillus rhamnosus* [102] are associated with protection from developing asthma in later life.

**Fig. 2:**
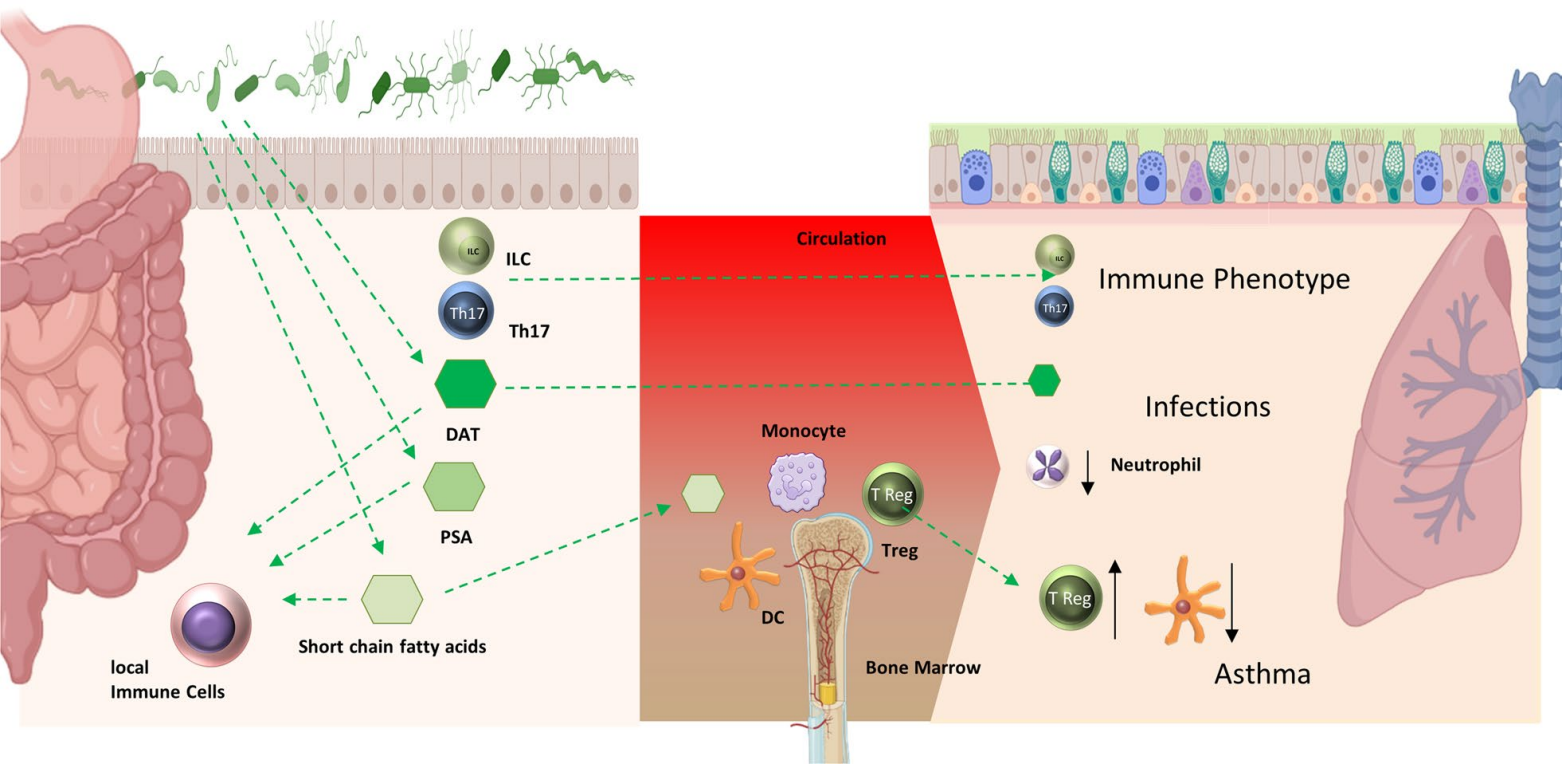
Intestinal/lung microbial axis. In the intestine, the microbiome communicates with structural and immune cells of the host via the release of microbial antigens, TLR ligands or metabolites such as SCFA or deaminotyrosine (DAT). In this process, a kind of immune system fine-tuning occurs, supporting the symbiotic community between bacteria and host. Anti-inflammatory metabolites can also enter the circulation and influence immune responses in distal parts of the body. In addition to the systemic release of metabolites, the migration of cells from the intestine to the periphery and their immunoregulatory function are also shown. This can be anti-inflammatory, but also support processes that are needed to defend against infections. Picture adapted from: Wypych TP et al. [106]

Similar to other organs, SCFA produced in the gastrointestinal tract that enter the bloodstream and therefore the systemic circulation are thought to be a central regulator in lung immunology. High fiber diets increase SCFA levels and protect against the development of allergic disease. Data from an epidemiological study showed that higher stool concentrations of SCFA in early life were associated with reduced susceptibility to the development of atopic diseases in later life [103]. In an animal model of asthma, oral application of SCFA reduced the developing asthma phenotype by increasing the percentage of Tregs [103]. Moreover, SCFA reduce the survival and mobility of human eosinophils. SCFA-dependent reduction of eosinophils contributed to amelioration of the asthmatic phenotype in mice [104]. SCFA also appear to affect monocyte and subsequent DC macrophage development towards increased phagocytosis but reduced T cell activation ability [105]. Cait et al. reported that dysbiosis of SCFA-producing gut bacteria can affect systemic DC and T cell responses and thereby modulate allergic lung inflammation. Treatment with SCFA reduced the ability to mount enhanced antigen-specific adaptive immune responses and ameliorated lung disease [107] (Fig. 2). The SCFA butyrate is also able to reduce activation of murine and human innate lymphoid cells type 2 [ILC2] [108]. These cells are involved in innate immunity and can produce IL-5 and IL-13, similar to Th2 cells. However, in contrast to their T cell counterparts, they seem to be resistant to treatment with corticosteroids, and are therefore associated with severe eosinophilic asthma. Interestingly, diet-mediated induction of SCFA reduced the capability of murine ILC2 cells to induce lung inflammation [109]. Initial clinical trials show that supplementation with soluble fiber, to increase SCFA levels, improved asthma control and inflammation. Even if currently available data are limited by small sample sizes and short follow-up, it does provide and initial indication that diets inducing SCFA could be potential add-on treatment for asthma [110]. PSA has also had beneficial effects on asthma development. In a mouse model, gastrointestinal exposure to PSA derived from the commensal bacterium *Bacteroides fragilis* reduced susceptibility to develop asthma [73].

Gut-lung axis communication has also been reported to have negative consequences. Antibiotic-induced dysbiosis in the gut can lead to an overgrowth of microbiota-associated fungi. These fungi, mainly belonging to *Candida* species, induce inflammatory responses. Release of mediators like prostaglandin E2 can shape circulating monocytes towards M2 macrophages and these are able to exacerbate lung inflammation [111]. Likewise, expansion of the commensal fungus *Wallemia mellicola* has been linked to the severity of asthma. Mice colonized with the fungus demonstrated increased signs of asthma, like airway hyperresponsiveness, BAL eosinophilia or goblet cell metaplasia upon allergen challenge. The signs were associated with an increased secretion of allergen positive immunoglobulins and IL-13 producing T cells [112].

Administration of probiotics, prebiotics or synbiotics can help to maintain, restore or support a healthy gut microbiome and so strengthen the beneficial arm of the gut–lung axis. Especially when done in early life, this is thought to reduce susceptibility to asthma development. Based on observations that low abundances of *Lactobacilli* was associated with asthma risk, bacilli from this species were considered as potential probiotics and are still one of the most common probiotics [113]. Unfortunately, the effectiveness of such treatments to attenuate or prevent asthma in humans are so far not convincing. However, animal data regarding the usage of probiotics as therapeutic intervention for allergic airway disease is promising.

Application of probiotics like *Lactobacillus rhamnosus* [114, 115], *Lactobacillus reuteri* [116], *Lactobacillus gasseri*, [117], and *Bifidobacterium infantis* [118] all reduced the development of allergic airway diseases in mice. Treatment was associated with induction of Tregs and modulation of the ratio T helper cell subtypes. The effectiveness of *Lactobacillus Rhamnosus* was shown in both chronic prophylactic and therapeutic models of allergic airway disease in mice [119].

Prebiotics have also been investigated as potential add-on treatment for asthma. Administration of the prebiotic mannose receptor blocker “mannan”, derived from *Saccharomyces cerevisiae,* had beneficial effects on airway inflammation and remodeling in a murine asthma model [120]. Interestingly, mannan seems to be also involved in human epithelial repair processes [120]. Prophylactic immune dampening effectiveness of probiotic *Lactobacillus Rhamnosus* and of a prebiotic crude turmeric extract were also observed in a house dust mite-specific murine asthma model, and symbiotic application of bacterium and extract improved therapeutic effectiveness [121]. Similar results have been obtained after application of long-chain fructooligosaccharide (lcFOS) combined with *Bifidobaterium breve* M-16 V [122, 123].

Meta-analysis of clinical data showed beneficial effects of probiotics to reduce susceptibility to develop eczema in later life, there was no indication of their effectiveness for the treatment for wheezing or preventing asthma development in children [124–126]. However, there are some positive studies. Van der Aa reported a reduction in respiratory symptoms in children receiving a symbiotic formula consisting of a hydrolyzed *Bifidobacterium breve M-16 V* altogether galacto/fructooligosaccharide mixture [127].

Clinical trials in adults are scarce. One small, short-term trial reported improvements in airway inflammation and asthma control after the application of prebiotics, but the authors recommended the need for larger-scale trials to confirm the potential of fiber diets an addition to asthma management [110]. In a review summarizing the effectiveness of *Lactobacillus* species in allergic rhinitis, Steiner and Lorentz presented therapeutic effectiveness from murine experiments and human trials [128]. They highlighted an interaction between *Lactobacillus* and the immune system, and noted that the majority of clinical trials showed beneficial effects. Nevertheless, they also noted that further studies are needed to provide precise information concerning appropriate species, dosage and timing of treatment, and to facilitate understanding of the underlying mechanism(s) of any benefit.

## Environmental factors derived from exogenous microbiota offer protection from atopy

Exogeneous stressors like allergens, pollutants (e.g. cigarette smoke) or pathogens altered microbiome composition and thus contribute to the development of lung diseases [99]. Today we also know that exogeneous “relaxators” exist, and that these can be beneficial microbes, microbial-derived components or proteins exerting protective effects. Differences in microbial communities, their components and metabolites between urban and rural and rural farming sides have been discussed as significantly contributing to protection against asthma [129–135]. Contact with exogenous factors derived from countryside microbes in early life supports the development of a “healthy immune system”, whereas insufficient signals provided in urban sites leads to an inadequately trained immune system that can induce inappropriate responses and therefore increase susceptibility to develop allergies. Communication between microbes and host, and the shaping of a protective immune system, starts even before birth. For example, maternal exposure to a farm microbiota was associated with decreased asthma risk in offspring [136–139]. Comparing dust from rural and suburban areas in Germany, Ege et al. found a negative association between both gram-positive (staphylococci, corynebacteria, lactic acid fermenters) and gram-negative bacteria (neisseriae, Acinetobacter) and the development of asthma [140]. Beneficial effects of *Staphylococcus sciuri W620* (*S. sciuri W620*) could be confirmed in murine models of asthma [141].

The importance of microbial composition in protective effects was highlighted in another study, where children living in non-farm homes were protected from asthma development when their home dust microbiota was similar to a farm microbiota [142]. Likewise, living in close proximity to farms and access to raw cow’s milk reduced asthma susceptibility in later life in another study [143]. Components in farm dust [130] and cow’s milk [133] have been identified as mediators of immune protective functions. The Pasteur study followed 1133 children from rural areas from age 0 to 6 and identified that continuous consumption of unprocessed cow’s milk was associated with increased Treg numbers and a reduced susceptibility to develop asthma in later life [41]. This finding could be partly explained by a higher uptake of omega-3 polyunsaturated fatty acids in unprocessed cow’s milk [144].

These findings are not intended as a recommendation to consume raw cow's milk directly, as this is associated with a number of foodborne illnesses. Rather, it is important to identify beneficial components of milk and make them available to humans in a safe form as a medicine or dietary supplement.

Deciphering underlying components, LPS concentrations in dust were associated with a reduced susceptibility to develop asthma in children growing up on a farm [145]. Mouse models confirmed that farm dust is a strong immune modulator and can prevent the development of asthma in mice [146]. Interestingly, not all farms seem to have protective properties. Further epidemiological studies revealed that the type of farm is an important modulator for the mediation of ignorance towards “harmless” environmental antigens [147]. Especially cattle and pig farms, but not farms keeping animals like hares, rabbits or sheep, had a protective effect. A study analyzed asthma development of Amish and Hutterite children, both with similar genetic ancestries and farming lifestyle, and found that Hutterite children were particularly prone to develop atopy and asthma in later life [148]. The main differences between the two communities is the technological level of farming, with the Amish using more traditional methods and the Hutterite using more advanced methods. This results in differences in the composition of stable and household dusts. Higher endotoxin levels in Amish dust were associated with differences in the modulation of innate immune cell activation towards tolerance induction. Moreover, animals receiving Amish, but not Hutterite, dust demonstrated a reduced capability to develop an asthma phenotype.

It is important to note that the time of contact, the formulation and the dose of the endotoxin have an important influence on its mode of action. Various studies have also shown that LPS plays an important role in the development [149, 150] and exacerbation of lung diseases [151–155].

Interactions between the dust, structural epithelial cells and immune cells contribute to protective effects. Hammad et al. found that environmental factors like farm dust or chronic exposure to low concentrations of LPS can affect the threshold of allergen recognition by suppressing activation of epithelial cells and DC [156]. Epithelial cells seemed to mediate asthma protection via a mechanism that depends on the ubiquitin-modifying enzyme A20 [157]. Clinical trials confirmed the immune regulatory association of TNF-α-induced protein 3 (TNFAIP3; A20) and asthma in humans. Treatment of PBMC derived from rural asthmatics with farm dust restored TNFAIP3 to levels comparable to those in healthy individuals and induced an anti-inflammatory state [158]. Farm dust also increased barrier function of epithelial cells, and this was associated with a reduction in viral uptake [159]. Since viral infections are associated with induction and exacerbation of asthma, this dust-mediated strengthening of barrier integrity might also have beneficial effects on asthma development and progression. Non-microbial substances in farm dust, such as N-glycolylneuraminic acid (Neu5Gc) [160], a glycoprotein expressed by non-human/non-bacterial cells, or Beta-lactoglobulin (a bovine-lipocalin), or plant-associated arabinogalactans [161], are also able to mediate immune protection [162].

## Old companions—new foes or still friends?

Growing knowledge about the interaction between the environment, microbiota and immune system has resulted in a revision of the hygiene hypothesis.

Industrialization and accompanying improvements in hygiene standards changed the make-up of our microbiota. Environmental stressors including pollutants/toxins, drugs (especially antibiotics), increased indoor and water hygiene standards, along with new approaches in childbirth and early childcare (Caesarean section; bottle-feeding) have had a large impact on the composition and ratios of microbes that have co-evolved with and in us. There has been a progressive loss of microbial species over several decades, which has had unforeseen consequences [163].

Today, many ancestral indigenous microbes like various bacteria (e.g. *Helicobacter pylori*), helminths and protozoa have been lost and are even been seen as pathogens. Based on available information, it can be assumed that this disappearance of microbes previously belonging to the host microbiome (and their compounds and metabolites) has an important impact on immunity and, subsequently, disease susceptibility. Having coevolved over thousands of years, microbes have developed inter-kingdom communication with the host that often has beneficial effects for both partners.

Next, we will review data relating to bacteria and helminths that were once associated with the human microbiome but have now largely been eliminated, especially in the industrialized world. Both beneficial and pathologic effects will be discussed.

## *Helicobacter pylori*

A textbook example of how the disappearance of ancestral bacteria can affect immunity and disease development is the gram-negative flagellated bacteria *Helicobacter pylori*. *H. pylori* can be regarded as one of the microbial companions of humans [164]. Colonizing as a dominant species in large numbers in a specific organ (the stomach) [165] over 58,000 years, *H. pylori* was once omnipresent in all humans. *H. pylori* colonizes the human stomach in youth and if not eradicated, persist through lifetime [166]. Today, approximately 50% of the world population is infected with the bacterium, but colonization rates are lowest in industrialized countries and highest in developing countries [167].

*H. pylori* developed several tactics to evade the immune system and protect itself against gastric acid [168]. Using its flagella [169] and following chemotactic signals [170, 171], the bacterium colonizes the mucus layer in the stomach. Moreover, *H. pylori* seemed to be masked against detection by pathogen recognition receptors because infections lead to an attenuated activation of adaptive immunity [172, 173]. Such interactions between *H. pylori* and adaptive immunity are of central importance for the development of immunological tolerance towards the bacterium.

Infection with *H. pylori* can lead to both pro- and anti-inflammatory immune reactions. It induces Tregs as well as Th1 and Th17 cells, along with the cytokines IFN-γ, IL-17 and TNF-α [174]. Neutrophils and monocytes support the development of these T cell responses, while Th17 cells induce the release of IL-8 and thus promote the neutrophil-mediated clearance of *H. pylori* [175]. In particular, exuberant and chronic inflammatory responses enhanced by environmental factors are responsible for a *H. pylori* gastric pathology resulting in peptic ulcer, primary gastric B cell lymphoma and gastric carcinoma. Details concerning the role of *H. pylori* in the development of these diseases are beyond the scope of this review and can be found elsewhere [176, 177].

The induction of Tregs is more likely to be associated with anti-inflammatory processes. Infections with *H. pylori* are associated with an induction of Tregs [178]. Naturally occurring Tregs and TGF-β seem to be particularly important for *H. pylori* colonization [179]. Interestingly, the depletion of Tregs not only led to decreased colonization with *H. pylori* but also to an increased inflammatory reaction [180]. Owyang et al. reported that TGF-β-producing DC play a central role in colonization and in *H. pylori*-mediated Treg immunology [181]. Interestingly, the induction of Tregs and the associated increased H. pylori colonization also seems to be also involved in the progression of gastric tumors [182].

**Fig. 3:**
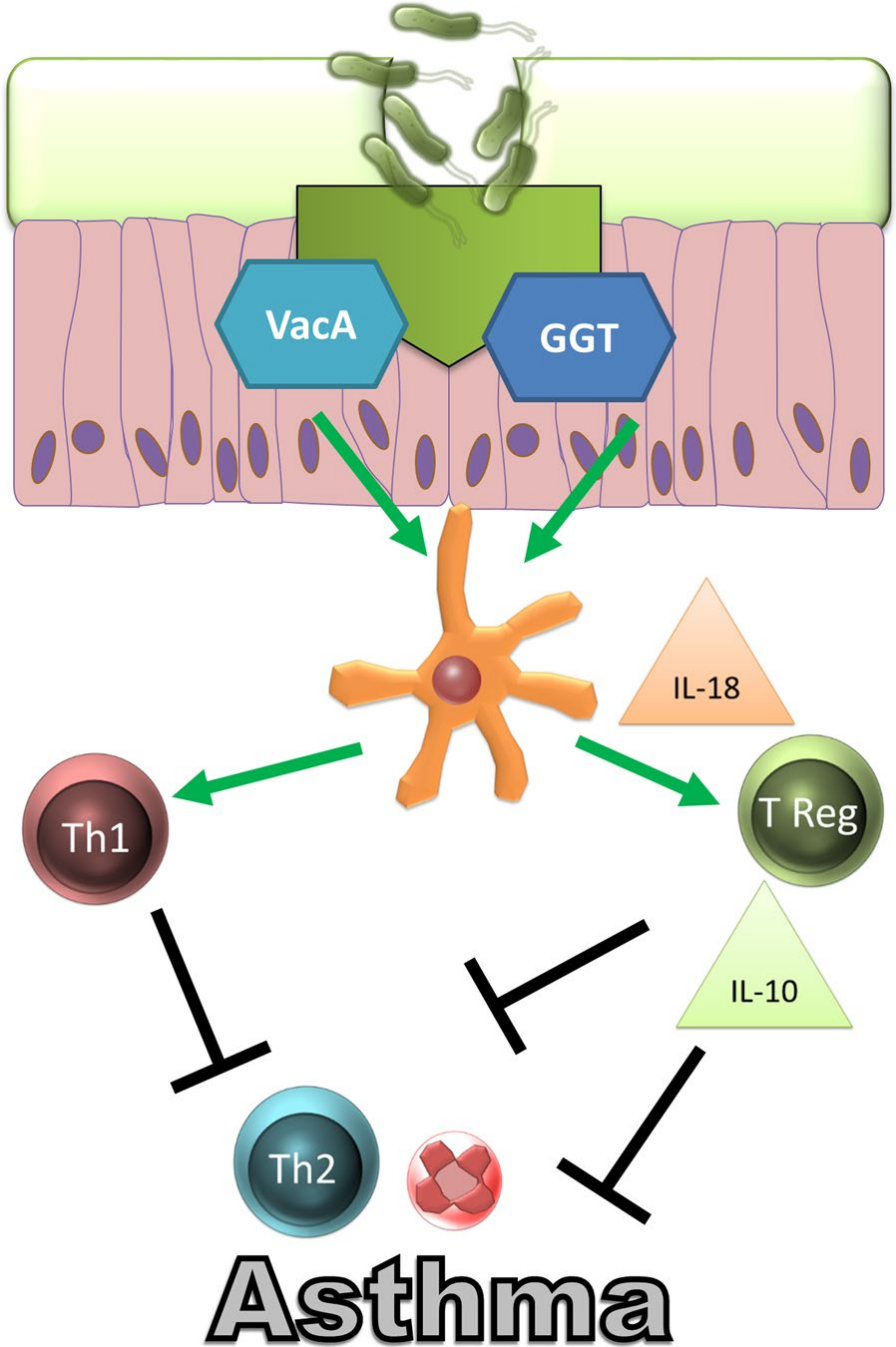
*Helicobacter pylori* can act as an immunoregulator via VacA and GGT. Both proteins induce a tolerogenic DC phenotype that can induce Tregs or Th1 cells. Among other things, the T cells have an anti-inflammatory effect via the release of IL-10 and can thus suppress the development of an allergic respiratory disease

Virulence factors, like cytotoxin-associated gene A (CagA), vacuolating cytotoxin A (VacA), γ-glutamyl transferase (GGT), neutrophil-activating protein (HP-NAP) and adhesins are interaction factors that help the bacterium to attach and communicate with the host. The functionality of these factors depends on the strain and can therefore differentially contribute to pro-, but also anti-inflammatory, *H. pylori*-driven host responses. Amedia et al. showed that neutrophils and monocytes produce IL-12 in response to HP-NAP and are thus able to induce IFN-γ-driven Th1 gastric inflammation [183]. Arginin [184], VacA and GGT dampen T cell responses and therefore support the survival of the bacteria. VacA is able to directly suppress bacterial proliferation [185] and modulate activation. Effects seem to be mediated by VacA binding to CD18 [186]. Likewise, GGT mediates T cell suppression by the induction of cell cycle arrest [187]. Both, GGT and VacA induced DC-dependent Tregs and suppressed the activity of CD4^+^ T cells [188] (Fig. 3).

Overall, *H. pylori* infections modulate host immunity, resulting in both pro-inflammatory and anti-inflammatory responses that on the one hand affect bacterial colonization and development of gastric diseases but on the other hand have the potential to orchestrate protective immunity that is capable of suppressing misguided immune responses that otherwise result in diseases like allergy.

Several epidemiological studies support the hypothesis for the beneficial role of this, once commensal, bacteria. These show that colonization with *H. pylori* in early childhood is negatively associated with the development asthma [189]. Further cross-sectional studies and meta-analyses confirmed this observation and reported an inverse association between *H. pylori* infections and the development of asthma in children and adults [190–198]. In particular, CagA positive strains [196, 198, 199] and maternal *H. pylori* status [200] seem to influence the susceptibility to asthma development. However, several studies failed to find an inverse relationship between *H. pylori* infection and asthma development, or had inconclusive results [201–205].This highlighted the need for additional studies to investigate *H. pylori*-host interactions.

A decade ago, the first studies using a murine model began to examine the role of *H. pylori* infections in the development of asthma in more detail. Isabelle Arnold showed that neonatal animals infected with *H. pylori* had an attenuated asthma phenotype in later life [206]. Transfer experiments found that Tregs played an important role in the *H. pylori*-mediated immune protection [206]. Further work by the same research group showed that *H. pylori* modulates DC and that these are involved in the development of immunoprotective Tregs via the release of IL-18 [207]. DC infected with *H. pylori* mutants devoid of virulence factors VacA or GGT failed to generate tolerogenic DC and immune protection, indicating a central role for both of these factors in *H. pylori*-mediated protection from asthma development [188]. The above data confirmed the epidemiological studies and showed that postnatal infection with *H. pylori* protected against the development of asthma in later life.

The data also suggest that the administration of *H. pylori* could be suitable as a therapeutic strategy for the treatment of allergic diseases such as asthma. To avoid side effects of a live infection, experiments were carried out with bacterial extract. Comparable to live infections, prophylactic application of *H. pylori*-derived bacterial extracts modulated DC and Treg responses in a IL-10 dependent manner and attenuated the development of allergic airway disease in later life [208]. Similar to the clinical studies from den Hollander [200], trans maternal-induced asthma protection was also seen in mice. The offspring of mothers receiving bacterial extract during pregnancy plus during lactation showed fewer asthma signs in later life [209]. Interestingly, studies of therapeutic approaches also found that adult mice developed fewer signs of asthma like allergen induced airway inflammation and mucus secretion after treatment with *H. pylori* extract [210, 211].

Studies that applying purified VacA prophylactically after birth or by means of trans maternal transfer showed a protective effect [208, 209]. Moreover, recently published studies have shown that VacA is also therapeutically effective. In acute or therapeutic murine models of allergic airway disease [212], including a chronic disease model [213], treatment with VacA attenuated airway disease. Similar to the prophylactic models, induction of Tregs was observed. In addition, repeated treatment with VacA in the chronic model appeared to suppress the development of the local lung-specific adaptive immunological memory. VacA affects myeloid cells in the gastric mucosa creating a Treg-inducing tolerogenic milieu [214]. These cells are capable of migrating within the body and thereby mediating immune suppression. This in turn could reduce the capability of mounting excessive immune responses and thus reduce susceptibility to develop allergies.

In addition to VacA, other *H. pylori*-derived molecules have been reported to mediate immune suppression. Zhou et al. showed that recombinant *H. pylori* NAP (rNAP) suppressed ovalbumin-induced allergic airway disease in mice in a prophylactic manner [215].

Currently available data indicate that *H. pylori* is an indigenous commensal microbe that co-evolved with humans. During a cohabitation period of approximately 60,000 years, the development of communication between host and bacteria has resulted in immune dampening effects in the host, which allow colonization and survival of the bacteria. Improved hygiene standards led to the disappearance of the bacteria and thus presumably also to a change in immune responsiveness that has contributed to the development of allergic diseases. Deciphering the protective mechanisms could provide the tools needed to help avoid and treat allergic disease such as asthma. It is important to note that potential side effects of H. pylori already discussed are excluded and only the beneficial properties of the bacterium are identified.

## Helminths

Like bacteria such as *H. pylori,* intestinal parasites also co-evolved with humans and parasitic infections still affect 2 million people worldwide, especially in developing countries [200]. Of these, protozoa and helminths are of central importance for human health [216]. Co-evolutionary acquired mechanisms allow helminths suppress host defense mechanisms and these organisms remain in the host for up to 20 years [217]. The naturally occurring immune response against helminths is a pronounced type 2 response, phenotypically similar to an allergic immune reaction.

The observations led to the concept that both, anti-inflammatory endogenous processes to restore homeostasis after strong Th2 responses to worm infections, but also escape mechanisms developed by the parasite contribute to asthma protection. In particular, chronic (but not acute) helminth infections seem able to create regulatory environments capable of suppressing immune responses to harmless antigens/allergens [218].

One of the first clinical studies analyzing the relationship between helminth infection and the development of allergy made two key observations. It found that children infected with *Schistosoma haematobium* had a lower prevalence of HDM allergies, and that there was a correlation between the reduction of HDM-specific antibodies and helminth-specific induction of the anti-inflammatory cytokine IL-10 [219]. Subsequently, numerous other studies also showed an inverse correlation between helminth infections and the development of allergies [220–224]. Again, however, published data are not consistent, with other studies finding a positive correlation or no correlation at all [225–228].

Epidemiological studies suggest that the influence of helminths on asthma is strongly dependent on the helminth species and the time, duration and strength of infection [229, 230]. Smits and colleagues summarized these relationships very well [218]. They emphasized that early childhood and chronic infections in particular have protective effects. Infections with high numbers of parasites seem to have an immune-protective effect, whereas weak infection processes are more likely to be associated with the development of allergies. Regarding the helminth species, infections with trichuris, hookworm, or schistosome protect from the development of allergies, while infections with *Ascaris lumbricoides* [231–233] and especially worms for which humans are not normally the host (*Toxocara spp*) [234], are positively associated with the development of atopy. Clear identification of protective species and immune-dampening molecules could provide new therapeutic approaches for the treatment of allergic diseases.

**Fig. 4:**
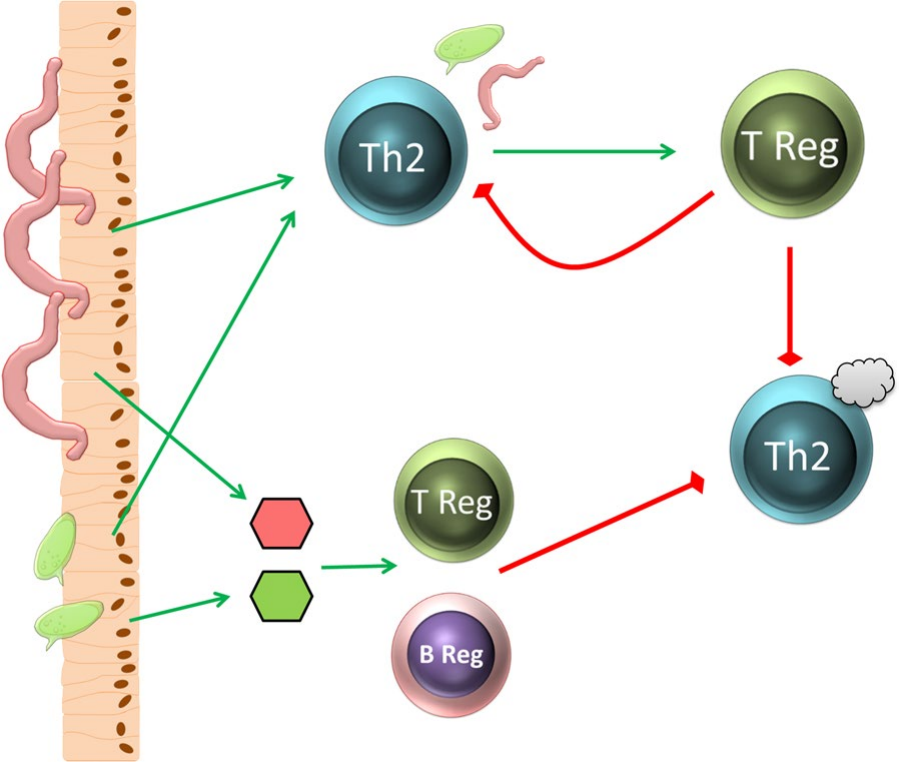
Comparable to allergy, strong Th2 immune responses are induced to eliminate worms. Especially for human-associated worms, infection also leads to the development of an anti-inflammatory immune response in which both anti-inflammatory T and B cells are induced. It is believed that both cell types, which can be induced by the worm itself and by components from its eggs, can prevent the development of allergies

Animal models helped to clarify the immune regulatory role of helminth infection and allergic diseases. Moreover, they provided the first data about the therapeutic effectiveness of immune-suppressive helminth-derived molecules [235]. Worms belonging to the species Schistosoma in particular showed promising effects. Chronic infection with *Schistosoma mansoni* resulted in an immune regulatory milieu capable of suppressing the development of allergic airway diseases [229, 236]. Transfer experiments found that T cells and B cells are important in the mediation of this immune suppression [229]. Comparable results were observed in mice infected with *Schistosoma japonicum* [237], and DC also appear to play an important role in the protective effects of helminth infection [238]. Transfer of DC isolated from helminth-infected mice enhanced Treg responses in airway allergic inflammation [239]. Interestingly, in worm infections, regulatory B cells also appear to have an important function in mediating the immune suppressive effects [240] (Fig. 4).

Other studies suggest that worm eggs play a central role for Treg induction and are therefore beneficial for the suppression of allergies [241, 242]. Prophylactic treatment of mice with eggs derived from *Schistosoma mansoni* attenuated allergic airway disease; the effects were independent of B cells and Tregs but were associated with a strong systemic helminth egg-specific Th2 response [243]. Application of eggs can also result in strong lung inflammation accompanied by granuloma formation [244]. Because of these side effects, efforts have been made to identify components of worms and their eggs that induce protective but not inflammatory processes and thus could be suitable as potential therapeutic agents [245]. Initial studies showed that crude mixtures prepared from worms and eggs can attenuate the development of diseases, including type 1 diabetes [246, 247]. The mixture of antigens modulates both arms of immunity [248]. DCs and monocytes are differentially activated, and show a cytokine and costimulatory cytokine pattern that indicates the induction of both effector and regulatory T cell responses [248]. Ongoing projects identified different worm- or egg-derived molecules that were capable of suppressing the immune response and thereby attenuating the development of allergic airway diseases [249–251]. As previously mentioned with H. pylori, it is imperative to keep the safety aspect in mind when developing new therapeutic strategies based on the use of pathogen-associated molecules.

Worm colonization can lead to severe medical problems, especially in the chronic course. When researching new worm-based drugs, it is therefore important to exclude negative mechanisms of action and to identify and isolate as many positive aspects as possible and formulate them into an effective drug.

As well as plathelminthes like *Schistosoma,* nematodes have also shown beneficial effects for prevention of asthma. Live infections [252] and treatment with molecules derived from animals in this phylum [253] induced anti-inflammatory responses.

Taken together, currently available data suggests that worms, especially those that have humans as a natural host, have immune dampening effects. The induction of Tregs and B cells, and the release of the anti-inflammatory cytokine IL10 play a central role. Active proteins can be found in the worms themselves and their eggs. Targeted characterization of these proteins could provide new therapeutic options for the treatment of allergic diseases. Evans and Mitre have summarized the mouse models for different allergic diseases in which helminths show prophylactic or therapeutic benefit [235]. In addition, their review highlighted that infections are effective in mice, but that initial clinical studies in humans were largely unable to show any positive effects of treatment with worm components [235]. In 2020, Ryan et al. provided an update on the efficacy of helminth infection in clinical trials [254]. The article summarized data on the effectiveness of treatments with the pig whipworm *Trichuris suis* or the human hookworm *Necator americanus* in different inflammatory human diseases, including Crohn’s disease, ulcerative colitis, rheumatoid arthritis, multiple sclerosis, allergic rhinitis and asthma [254].

As with other microbes, data in this area are not consistent. Some studies have failed to find any effect of helminth infection, and results vary depending on the worm species and the clinical setting. Just like bacteria, worms have evolved with humans and have developed mechanisms that ensure the survival of both the host and the microorganism. The decoding of these mechanisms and the creation of target structures that are therapeutically effective are needed if worm-based therapeutics are to be developed and applied in the future.

## Conclusions

**Fig. 5:**
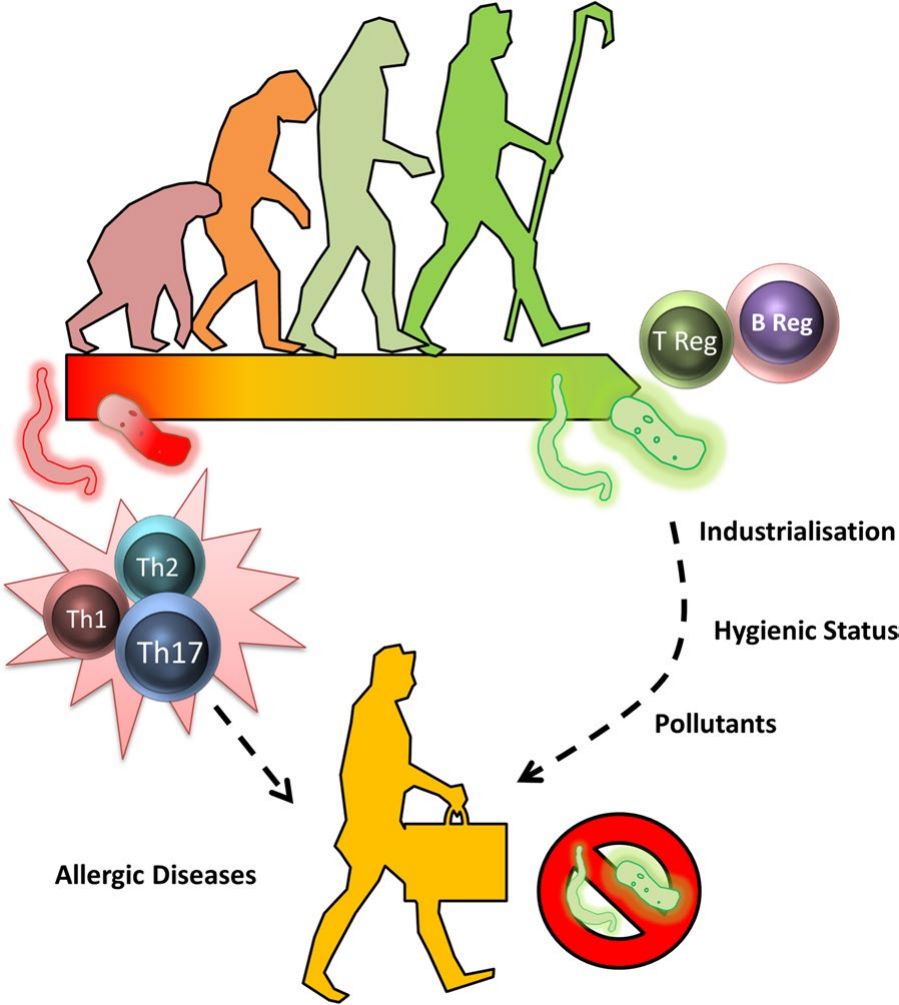
Humans have evolved together with a wide range of bacteria, viruses, worms and fungi. In this process, favorable communities have developed that have a positive effect on metabolism and the immune system, and a negative effect on the development of diseases. Alternations in this coexistence, such as changes in lifestyle (industrialization, hygiene status, pollutants) mean that modern man is more susceptible to the development of diseases such as allergies or asthma

Over time, humans have co-evolved with a large number of microbes that live on or in us. In this process, a community of life has evolved consisting of the microbiome, which includes bacteria, viruses and fungi, with humans as the host as seen in Fig. 5. The communication between the host and the microbiome has a significant impact on immunological and metabolic processes. Therefore, it is not surprising that disturbances in the microbiome can have an impact on the development and progression of diseases. In recent years, research into the interaction of the environment with the microbiome and the host has helped to identify processes that can have both positive and negative effects on our health. Targeting microbiome-associated health-promoting effects and avoiding the effects associated with disease development has the potential to contribute to the development of new therapeutic options in the coming years. These could function via direct manipulation of an existing microbiome using pre- or probiotics, or via the targeted use of specific beneficial microbial strains, their metabolites or individual components.

## Data Availability

Not applicable.

## Abbreviations

IL: Interleukin
TSLP: Thymic stromal lymphopoietin
Treg: Regulatory T cell
TGF: Transforming growth factor
DC: Dendritic cell
MALDI-TOF: Matrix-assisted laser desorption/ionization-time of flight
PAMPS: Pathogen-associated molecular patterns
AHR: Aryl hydrocarbon receptor
FMT: Fecal microbiome transplantations
COPD: Chronic obstructive pulmonary disease
TLR: Toll like receptor
NOD2: Nucleotide oligomerization domain 2
MHC: Major histocompatibility complex
IFN: Interferon
LTA: Lipoteichoic acid
PGN: Peptidoglycan
SCFA: Short chain fatty acid
Breg: Regulatory B cell
CFB: Cytophaga-flavobacter-bacter-oidetes
SFB: Segmented filamentous bacteria
DAT: Deaminotyrosine
ILC2: Lymphoid cells type 2
lcFOS: Long-chain fructooligosaccharide
TNFAIP3: TNF-α-induced protein 3
Neu5Gc: N-glycolylneuraminic acid
CagA: Cytotoxin-associated gene A
VacA: Vacuolating cytotoxin A
GGT: γ-Glutamyl transferase

## References

[1] The Global Asthma Report. Int J Tuberc Lung Dis 2022;26:S1-S102. 10.5588/ijtld.22.1010 36303302

[2] Kemp A: First and only biologic approved in the EU in patients with severe asthma with no phenotype or biomarker limitations. https://www.astrazeneca.com/media-centre/press-releases/2022/tezspire-approved-in-the-eu-for-the-treatment-of-severe-asthma.html#:~:text=Tezspire%20is%20the%20first%20and,TSLP)%2C%20an%20epithelial%20cytokine. 2022. Accessed 10 Jan 2024.

[3] Shevyrev D, Tereshchenko V (2020). Treg heterogeneity, function, and homeostasis. Front Immunol.

[4] Dehzad N, Bopp T, Reuter S, Klein M, Martin H, Ulges A (2011). Regulatory T cells more effectively suppress Th1-induced airway inflammation compared with Th2. J Immunol.

[5] Martin H, Reuter S, Dehzad N, Heinz A, Bellinghausen I, Saloga J (2012). CD4-mediated regulatory T-cell activation inhibits the development of disease in a humanized mouse model of allergic airway disease. J Allergy Clin Immunol.

[6] Jartti T, Bønnelykke K, Elenius V, Feleszko W (2020). Role of viruses in asthma. Semin Immunopathol.

[7] Strachan DP (1989). Hay fever, hygiene, and household size. BMJ.

[8] Sender R, Fuchs S, Milo R (2016). Revised estimates for the number of human and bacteria cells in the body. PLoS Biol.

[9] Belkaid Y, Hand TW (2014). Role of the microbiota in immunity and inflammation. Cell.

[10] Iacob S, Iacob DG, Luminos LM (2018). Intestinal microbiota as a host defense mechanism to infectious threats. Front Microbiol.

[11] Asnicar F, Berry SE, Valdes AM, Nguyen LH, Piccinno G, Drew DA (2021). Microbiome connections with host metabolism and habitual diet from 1,098 deeply phenotyped individuals. Nat Med.

[12] Bäckhed F, Ding H, Wang T, Hooper LV, Koh GY, Nagy A (2004). The gut microbiota as an environmental factor that regulates fat storage. Proc Natl Acad Sci U S A.

[13] Rowland I, Gibson G, Heinken A, Scott K, Swann J, Thiele I (2018). Gut microbiota functions: metabolism of nutrients and other food components. Eur J Nutr.

[14] Morais LH, Schreiber HL, Mazmanian SK (2021). The gut microbiota–brain axis in behaviour and brain disorders. Nat Rev Microbiol.

[15] Schroeder BO, Bäckhed F (2016). Signals from the gut microbiota to distant organs in physiology and disease. Nat Med.

[16] Mills S, Shanahan F, Stanton C, Hill C, Coffey A, Ross RP (2013). Movers and shakers. Gut Microbes.

[17] Citorik RJ, Mimee M, Lu TK (2014). Sequence-specific antimicrobials using efficiently delivered RNA-guided nucleases. Nat Biotechnol.

[18] Liu SX, Li YH, Dai WK, Li XS, Qiu CZ, Ruan ML (2017). Fecal microbiota transplantation induces remission of infantile allergic colitis through gut microbiota re-establishment. World J Gastroenterol.

[19] Hwang IY, Koh E, Wong A, March JC, Bentley WE, Lee YS (2017). Engineered probiotic *Escherichia coli* can eliminate and prevent *Pseudomonas aeruginosa* gut infection in animal models. Nat Commun.

[20] Börner RA, Kandasamy V, Axelsen AM, Nielsen AT, Bosma EF (2019). Genome editing of lactic acid bacteria: opportunities for food, feed, pharma and biotech. FEMS Microbiol Lett.

[21] Turnbaugh PJ, Ley RE, Hamady M, Fraser-Liggett CM, Knight R, Gordon JI (2007). The human microbiome project. Nature.

[22] Segal LN, Alekseyenko AV, Clemente JC, Kulkarni R, Wu B, Chen H (2013). Enrichment of lung microbiome with supraglottic taxa is associated with increased pulmonary inflammation. Microbiome.

[23] Charlson ES, Bittinger K, Haas AR, Fitzgerald AS, Frank I, Yadav A (2011). Topographical continuity of bacterial populations in the healthy human respiratory tract. Am J Respir Crit Care Med.

[24] Noval Rivas M, Crother TR, Arditi M (2016). The microbiome in asthma. Curr Opin Pediatr.

[25] Dickson RP, Erb-Downward JR, Freeman CM, McCloskey L, Falkowski NR, Huffnagle GB (2017). Bacterial topography of the healthy human lower respiratory tract. MBio.

[26] Hilty M, Burke C, Pedro H, Cardenas P, Bush A, Bossley C (2010). Disordered microbial communities in asthmatic airways. PLoS ONE.

[27] Pang Z, Wang G, Gibson P, Guan X, Zhang W, Zheng R (2019). Airway microbiome in different inflammatory phenotypes of asthma: a cross-sectional study in Northeast China. Int J Med Sci.

[28] Proctor LM, Creasy HH, Fettweis JM, Lloyd-Price J, Mahurkar A, Zhou W (2019). The integrative human microbiome project. Nature.

[29] Pérez-Losada M, Authelet KJ, Hoptay CE, Kwak C, Crandall KA, Freishtat RJ (2018). Pediatric asthma comprises different phenotypic clusters with unique nasal microbiotas. Microbiome.

[30] Santee CA, Nagalingam NA, Faruqi AA, DeMuri GP, Gern JE, Wald ER (2016). Nasopharyngeal microbiota composition of children is related to the frequency of upper respiratory infection and acute sinusitis. Microbiome..

[31] Abdel-Aziz MI, Thorsen J, Hashimoto S, Vijverberg SJH, Neerincx AH, Brinkman P (2023). Oropharyngeal microbiota clusters in children with asthma or wheeze associate with allergy, blood transcriptomic immune pathways, and exacerbation risk. Am J Respir Crit Care Med.

[32] Marian GN, Laura M, Xavier P, Rafaela F, Vicente PB, Miguel G (2014). Severity-related changes of bronchial microbiome in chronic obstructive pulmonary disease. J Clin Microbiol.

[33] Boutin S, Graeber SY, Weitnauer M, Panitz J, Stahl M, Clausznitzer D (2015). Comparison of microbiomes from different niches of upper and lower airways in children and adolescents with cystic fibrosis. PLoS ONE.

[34] Herbst T, Sichelstiel A, Schär C, Yadava K, Bürki K, Cahenzli J (2011). Dysregulation of allergic airway inflammation in the absence of microbial colonization. Am J Respir Crit Care Med.

[35] Russell SL, Gold MJ, Hartmann M, Willing BP, Thorson L, Wlodarska M (2012). Early life antibiotic-driven changes in microbiota enhance susceptibility to allergic asthma. EMBO Rep.

[36] Russell SL, Gold MJ, Willing BP, Thorson L, McNagny KM, Finlay BB (2013). Perinatal antibiotic treatment affects murine microbiota, immune responses and allergic asthma. Gut Microbes.

[37] Borbet TC, Pawline MB, Zhang X, Wipperman MF, Reuter S, Maher T (2022). Influence of the early-life gut microbiota on the immune responses to an inhaled allergen. Mucosal Immunol.

[38] Beigelman A, Srinivasan M, Goss CW, Wang J, Zhou Y, True K (2022). Azithromycin to prevent recurrent wheeze following severe respiratory syncytial virus bronchiolitis. NEJM Evid..

[39] Ukkonen RM, Renko M, Kuitunen I (2023). Azithromycin for acute bronchiolitis and wheezing episodes in children—a systematic review with meta-analysis. Pediatr Res.

[40] Bacharier LB, Guilbert TW, Mauger DT, Boehmer S, Beigelman A, Fitzpatrick AM (2015). Early administration of azithromycin and prevention of severe lower respiratory tract illnesses in preschool children with a history of such illnesses: a randomized clinical trial. JAMA.

[41] Marri PR, Stern DA, Wright AL, Billheimer D, Martinez FD (2013). Asthma-associated differences in microbial composition of induced sputum. J Allergy Clin Immunol.

[42] Huang YJ, Nelson CE, Brodie EL, Desantis TZ, Baek MS, Liu J (2011). Airway microbiota and bronchial hyperresponsiveness in patients with suboptimally controlled asthma. J Allergy Clin Immunol.

[43] Goleva E, Jackson LP, Harris JK, Robertson CE, Sutherland ER, Hall CF (2013). The effects of airway microbiome on corticosteroid responsiveness in asthma. Am J Respir Crit Care Med.

[44] Zhang Q, Cox M, Liang Z, Brinkmann F, Cardenas PA, Duff R (2016). Airway microbiota in severe asthma and relationship to asthma severity and phenotypes. PLoS ONE.

[45] Perdijk O, Marsland BJ (2019). The microbiome: toward preventing allergies and asthma by nutritional intervention. Curr Opin Immunol.

[46] Sevelsted A, Stokholm J, Bønnelykke K, Bisgaard H (2015). Cesarean section and chronic immune disorders. Pediatrics.

[47] Thavagnanam S, Fleming J, Bromley A, Shields MD, Cardwell CR (2008). A meta-analysis of the association between Caesarean section and childhood asthma. Clin Exp Allergy.

[48] Ahmadizar F, Vijverberg SJH, Arets HGM, de Boer A, Garssen J, Kraneveld AD (2017). Breastfeeding is associated with a decreased risk of childhood asthma exacerbations later in life. Pediatr Allergy Immunol.

[49] Round JL, Mazmanian SK (2009). The gut microbiota shapes intestinal immune responses during health and disease. Nat Rev Immunol.

[50] McDermott AJ, Huffnagle GB (2014). The microbiome and regulation of mucosal immunity. Immunology.

[51] Kopitar AN, Ihan Hren N, Ihan A (2006). Commensal oral bacteria antigens prime human dendritic cells to induce Th1, Th2 or Treg differentiation. Oral Microbiol Immunol.

[52] Stagg AJ (2018). Intestinal dendritic cells in health and gut inflammation. Front Immunol.

[53] Biedermann T, Skabytska Y, Kaesler S, Volz T (2015). Regulation of T cell immunity in atopic dermatitis by microbes: the yin and yang of cutaneous inflammation. Front Immunol.

[54] Kwon HK, Lee CG, So JS, Chae CS, Hwang JS, Sahoo A (2010). Generation of regulatory dendritic cells and CD4+Foxp3+ T cells by probiotics administration suppresses immune disorders. Proc Natl Acad Sci.

[55] Thye AYK, Law JWF, Tan LTH, Thurairajasingam S, Chan KG, Letchumanan V (2022). Exploring the gut microbiome in myasthenia gravis. Nutrients.

[56] Khazaie K, Zadeh M, Khan MW, Bere P, Gounari F, Dennis K (2012). Abating colon cancer polyposis by Lactobacillus acidophilus deficient in lipoteichoic acid. Proc Natl Acad Sci U S A.

[57] Mohamadzadeh M, Pfeiler EA, Brown JB, Zadeh M, Gramarossa M, Managlia E (2011). Regulation of induced colonic inflammation by Lactobacillus acidophilus deficient in lipoteichoic acid. Proc Natl Acad Sci.

[58] Nilsen NJ, Deininger S, Nonstad U, Skjeldal F, Husebye H, Rodionov D (2008). Cellular trafficking of lipoteichoic acid and Toll-like receptor 2 in relation to signaling; role of CD14 and CD36. J Leukoc Biol.

[59] Chang HC, Lin KH, Tai YT, Chen JT, Chen RM (2010). Lipoteichoic acid-induced TNF-α and IL-6 gene expressions and oxidative stress production in macrophages are suppressed by ketamine through downregulating Toll-like receptor 2-mediated activation oF ERK1/2 and NFκB. Shock.

[60] Kang SS, Ryu YH, Baik JE, Yun CH, Lee K, Chung DK (2011). Lipoteichoic acid from Lactobacillus plantarum induces nitric oxide production in the presence of interferon-γ in murine macrophages. Mol Immunol.

[61] Macho Fernandez E, Valenti V, Rockel C, Hermann C, Pot B, Boneca IG (2011). Anti-inflammatory capacity of selected lactobacilli in experimental colitis is driven by NOD2-mediated recognition of a specific peptidoglycan-derived muropeptide. Gut.

[62] Jeon SG, Kayama H, Ueda Y, Takahashi T, Asahara T, Tsuji H (2012). Probiotic Bifidobacterium breve induces IL-10-producing Tr1 cells in the colon. PLOS Pathog.

[63] Uribe-Herranz M, Rafail S, Beghi S, Gil-de-Gómez L, Verginadis I, Bittinger K (2020). Gut microbiota modulate dendritic cell antigen presentation and radiotherapy-induced antitumor immune response. J Clin Invest.

[64] Alameddine J, Godefroy E, Papargyris L, Sarrabayrouse G, Tabiasco J, Bridonneau C (2019). Faecalibacterium prausnitzii skews human DC to prime IL10-producing T cells through TLR2/6/JNK signaling and IL-10, IL-27, CD39, and IDO-1 induction. Front Immunol.

[65] Smith PM, Howitt MR, Panikov N, Michaud M, Gallini CA, Bohlooly-Y M (2013). The microbial metabolites, short-chain fatty acids, regulate colonic Treg cell homeostasis. Science.

[66] Kim CH, Park J, Kim M (2014). Gut microbiota-derived short-chain fatty acids, T cells, and inflammation. Immune Netw.

[67] Arpaia N, Campbell C, Fan X, Dikiy S, van der Veeken J, deRoos P (2013). Metabolites produced by commensal bacteria promote peripheral regulatory T-cell generation. Nature.

[68] Park J, Kim M, Kang SG, Jannasch AH, Cooper B, Patterson J (2015). Short-chain fatty acids induce both effector and regulatory T cells by suppression of histone deacetylases and regulation of the mTOR–S6K pathway. Mucosal Immunol.

[69] Singh N, Gurav A, Sivaprakasam S, Brady E, Padia R, Shi H (2014). Activation of Gpr109a, receptor for niacin and the commensal metabolite butyrate, suppresses colonic inflammation and carcinogenesis. Immunity.

[70] Luu M, Pautz S, Kohl V, Singh R, Romero R, Lucas S (2019). The short-chain fatty acid pentanoate suppresses autoimmunity by modulating the metabolic-epigenetic crosstalk in lymphocytes. Nat Commun.

[71] Luu M, Monning H, Visekruna A (2020). Exploring the molecular mechanisms underlying the protective effects of microbial SCFAs on intestinal tolerance and food allergy. Front Immunol.

[72] Tan J, McKenzie C, Vuillermin PJ, Goverse G, Vinuesa CG, Mebius RE (2016). Dietary fiber and bacterial SCFA enhance oral tolerance and protect against food allergy through diverse cellular pathways. Cell Rep.

[73] Johnson JL, Jones MB, Cobb BA (2015). Bacterial capsular polysaccharide prevents the onset of asthma through T-cell activation. Glycobiology.

[74] Telesford KM, Yan W, Ochoa-Reparaz J, Pant A, Kircher C, Christy MA (2015). A commensal symbiotic factor derived from Bacteroides fragilis promotes human CD39+Foxp3+ T cells and Treg function. Gut Microbes.

[75] Alvarez CA, Jones MB, Hambor J, Cobb BA (2020). Characterization of polysaccharide A response reveals interferon responsive gene signature and immunomodulatory marker expression. Front Immunol.

[76] Ong JS, Taylor TD, Yong CC, Khoo BY, Sasidharan S, Choi SB (2020). Lactobacillus plantarum USM8613 aids in wound healing and suppresses Staphylococcus aureus infection at wound sites. Probiot Antimicrob Proteins.

[77] Ivanov II, Frutos RdL, Manel N, Yoshinaga K, Rifkin DB, Sartor RB (2008). Specific microbiota direct the differentiation of IL-17-producing T-helper cells in the mucosa of the small intestine. Cell Host Microbe.

[78] Goto Y, Panea C, Nakato G, Cebula A, Lee C, Diez MG (2014). Segmented filamentous bacteria antigens presented by intestinal dendritic cells drive mucosal Th17 cell differentiation. Immunity.

[79] Jin Y, Wan Y, Chen G, Chen L, Zhang MQ, Deng L (2014). Treg/IL-17 ratio and Treg differentiation in patients with COPD. PLoS ONE.

[80] Mo C, Zeng Z, Deng Q, Ding Y, Xiao R (2018). Imbalance between T helper 17 and regulatory T cell subsets plays a significant role in the pathogenesis of systemic sclerosis. Biomed Pharmacother.

[81] Cervilha DAB, Ito JT, Lourenço JD, Olivo CR, Saraiva-Romanholo BM, Volpini RA (2019). The Th17/Treg cytokine imbalance in chronic obstructive pulmonary disease exacerbation in an animal model of cigarette smoke exposure and lipopolysaccharide challenge association. Sci Rep.

[82] Ratajczak P, Janin A, Peffault de Latour R, Leboeuf C, Desveaux A, Keyvanfar K (2010). Th17/Treg ratio in human graft-versus-host disease. Blood.

[83] Ji L, Zhan Y, Hua F, Li F, Zou S, Wang W (2012). The ratio of Treg/Th17 cells correlates with the disease activity of primary immune thrombocytopenia. PLoS ONE.

[84] Zhu J, Liu X, Wang W, Ouyang X, Zheng W, Wang Q (2017). Altered expression of regulatory T and Th17 cells in murine bronchial asthma. Exp Ther Med.

[85] Kelly J, Kennedy P, Cryan J, Dinan T, Clarke G, Hyland N (2015). Breaking down the barriers: the gut microbiome, intestinal permeability and stress-related psychiatric disorders. Front Cell Neurosci.

[86] Tang WHW, Li DY, Hazen SL (2019). Dietary metabolism, the gut microbiome, and heart failure. Nat Rev Cardiol.

[87] Bingula R, Filaire M, Radosevic-Robin N, Bey M, Berthon JY, Bernalier-Donadille A (2017). Desired turbulence? Gut-lung axis, immunity, and lung cancer. J Oncol.

[88] Bergman EN (1990). Energy contributions of volatile fatty acids from the gastrointestinal tract in various species. Physiol Rev.

[89] Perry RJ, Peng L, Barry NA, Cline GW, Zhang D, Cardone RL (2016). Acetate mediates a microbiome–brain–β-cell axis to promote metabolic syndrome. Nature.

[90] De Vadder F, Kovatcheva-Datchary P, Goncalves D, Vinera J, Zitoun C, Duchampt A (2014). Microbiota-generated metabolites promote metabolic benefits via gut-brain neural circuits. Cell.

[91] Zelante T, Iannitti RG, Cunha C, De Luca A, Giovannini G, Pieraccini G (2013). Tryptophan catabolites from microbiota engage aryl hydrocarbon receptor and balance mucosal reactivity via interleukin-22. Immunity.

[92] Ridlon JM, Kang DJ, Hylemon PB, Bajaj JS (2014). Bile acids and the gut microbiome. Curr Opin Gastroenterol.

[93] Chambers ES, Preston T, Frost G, Morrison DJ (2018). Role of gut microbiota-generated short-chain fatty acids in metabolic and cardiovascular health. Curr Nutr Rep.

[94] Adolph TE, Grander C, Moschen AR, Tilg H (2018). Liver–microbiome axis in health and disease. Trends Immunol.

[95] Lundgren P, Thaiss CA (2020). The microbiome-adipose tissue axis in systemic metabolism. Am J Physiol Liver Physiol.

[96] Schächtle MA, Rosshart SP (2021). The microbiota-gut-brain axis in health and disease and its implications for translational research. Front Cell Neurosci.

[97] Stiemsma LT, Arrieta MC, Dimitriu PA, Cheng J, Thorson L, Lefebvre DL (2016). Shifts in Lachnospira and Clostridium sp. in the 3-month stool microbiome are associated with preschool age asthma. Clin Sci.

[98] Arrieta MC, Stiemsma LT, Dimitriu PA, Thorson L, Russell S, Yurist-Doutsch S (2015). Early infancy microbial and metabolic alterations affect risk of childhood asthma. Sci Transl Med..

[99] Hufnagl K, Pali-Schöll I, Roth-Walter F, Jensen-Jarolim E (2020). Dysbiosis of the gut and lung microbiome has a role in asthma. Semin Immunopathol.

[100] Arrieta MC, Sadarangani M, Brown EM, Russell SL, Nimmo M, Dean J (2016). A humanized microbiota mouse model of ovalbumin-induced lung inflammation. Gut Microbes.

[101] van Nimwegen FA, Penders J, Stobberingh EE, Postma DS, Koppelman GH, Kerkhof M (2011). Mode and place of delivery, gastrointestinal microbiota, and their influence on asthma and atopy. J Allergy Clin Immunol.

[102] Durack J, Kimes NE, Lin DL, Rauch M, McKean M, McCauley K (2018). Delayed gut microbiota development in high-risk for asthma infants is temporarily modifiable by Lactobacillus supplementation. Nat Commun.

[103] Roduit C, Frei R, Ferstl R, Loeliger S, Westermann P, Rhyner C (2019). High levels of butyrate and propionate in early life are associated with protection against atopy. Allergy.

[104] Theiler A, Bärnthaler T, Platzer W, Richtig G, Peinhaupt M, Rittchen S (2019). Butyrate ameliorates allergic airway inflammation by limiting eosinophil trafficking and survival. J Allergy Clin Immunol.

[105] Trompette A, Gollwitzer ES, Yadava K, Sichelstiel AK, Sprenger N, Ngom-Bru C (2014). Gut microbiota metabolism of dietary fiber influences allergic airway disease and hematopoiesis. Nat Med.

[106] Wypych TP, Wickramasinghe LC, Marsland BJ (2019). The influence of the microbiome on respiratory health. Nat Immunol.

[107] Cait A, Hughes MR, Antignano F, Cait J, Dimitriu PA, Maas KR (2018). Microbiome-driven allergic lung inflammation is ameliorated by short-chain fatty acids. Mucosal Immunol.

[108] Sepahi A, Liu Q, Friesen L, Kim CH (2021). Dietary fiber metabolites regulate innate lymphoid cell responses. Mucosal Immunol.

[109] Lewis G, Wang B, Shafiei Jahani P, Hurrell BP, Banie H, Aleman Muench GR (2019). Dietary fiber-induced microbial short chain fatty acids suppress ILC2-dependent airway inflammation. Front Immunol.

[110] McLoughlin R, Berthon BS, Rogers GB, Baines KJ, Leong LEX, Gibson PG (2019). Soluble fibre supplementation with and without a probiotic in adults with asthma: a 7-day randomised, double blind, three way cross-over trial. EBioMedicine.

[111] Kim YG, Udayanga KGS, Totsuka N, Weinberg JB, Núñez G, Shibuya A (2014). Gut dysbiosis promotes M2 macrophage polarization and allergic airway inflammation via fungi-induced PGE2. Cell Host Microbe.

[112] Skalski JH, Limon JJ, Sharma P, Gargus MD, Nguyen C, Tang J (2018). Expansion of commensal fungus Wallemia mellicola in the gastrointestinal mycobiota enhances the severity of allergic airway disease in mice. PLOS Pathog.

[113] Johansson MA, Sjögren YM, Persson JO, Nilsson C, Sverremark-Ekström E (2011). Early colonization with a group of lactobacilli decreases the risk for allergy at five years of age despite allergic heredity. PLoS ONE.

[114] Jang SO, Kim HJ, Kim YJ, Kang MJ, Kwon JW, Seo JH (2012). Asthma prevention by Lactobacillus rhamnosus in a mouse model is associated with CD4+CD25+Foxp3+ T cells. Allergy Asthma Immunol Res.

[115] Wu CT, Chen PJ, Lee YT, Ko JL, Lue KH (2016). Effects of immunomodulatory supplementation with Lactobacillus rhamnosus on airway inflammation in a mouse asthma model. J Microbiol Immunol Infect.

[116] Li L, Fang Z, Liu X, Hu W, Lu W, Lee YK (2020). Lactobacillus reuteri attenuated allergic inflammation induced by HDM in the mouse and modulated gut microbes. PLoS ONE.

[117] Jan RL, Yeh KC, Hsieh MH, Lin YL, Kao HF, Li PH (2012). Lactobacillus gasseri suppresses Th17 pro-inflammatory response and attenuates allergen-induced airway inflammation in a mouse model of allergic asthma. Br J Nutr.

[118] Wang W, Luo X, Zhang Q, He X, Zhang Z, Wang X (2020). Bifidobacterium infantis relieves allergic asthma in mice by regulating Th1/Th2. Med Sci Monit Int Med J Exp Clin Res.

[119] Wu CT, Lin FH, Lee YT, Ku MS, Lue KH (2019). Effect of Lactobacillus rhamnosus GG immunopathologic changes in chronic mouse asthma model. J Microbiol Immunol Infect.

[120] Lew DB, Michael CF, Overbeck T, Robinson WS, Rohman EL, Lehman JM (2017). Beneficial effects of prebiotic Saccharomyces cerevisiae Mannan on allergic asthma mouse models. J Immunol Res.

[121] Ghiamati Yazdi F, Zakeri A, van Ark I, Leusink-Muis T, Braber S, Soleimanian-Zad S (2020). Crude turmeric extract improves the suppressive effects of Lactobacillus rhamnosus GG on allergic inflammation in a murine model of house dust mite-induced asthma. Front Immunol.

[122] Vos AP, van Esch BC, Stahl B, M’Rabet L, Folkerts G, Nijkamp FP (2007). Dietary supplementation with specific oligosaccharide mixtures decreases parameters of allergic asthma in mice. Int Immunopharmacol.

[123] Sagar S, Vos AP, Morgan ME, Garssen J, Georgiou NA, Boon L (2014). The combination of Bifidobacterium breve with non-digestible oligosaccharides suppresses airway inflammation in a murine model for chronic asthma. Biochim Biophys Acta Mol Basis Dis.

[124] Zuccotti G, Meneghin F, Aceti A, Barone G, Callegari ML, Di Mauro A (2015). Probiotics for prevention of atopic diseases in infants: systematic review and meta-analysis. Allergy.

[125] Azad MB, Coneys JG, Kozyrskyj AL, Field CJ, Ramsey CD, Becker AB (2013). Probiotic supplementation during pregnancy or infancy for the prevention of asthma and wheeze: systematic review and meta-analysis. BMJ Br Med J.

[126] Lin J, Zhang Y, He C, Dai J (2018). Probiotics supplementation in children with asthma: a systematic review and meta-analysis. J Paediatr Child Health.

[127] van der Aa LB, van Aalderen WMC, Heymans HSA, Henk Sillevis Smitt J, Nauta AJ, Knippels LMJ, et al. Synbiotics prevent asthma-like symptoms in infants with atopic dermatitis. Allergy. 2011;66:170–7. 10.1111/j.1398-9995.2010.02416.x 20560907

[128] Steiner NC, Lorentz A (2021). Probiotic potential of lactobacillus species in allergic rhinitis. Int Arch Allergy Immunol.

[129] Riedler J, Braun-Fahrländer C, Eder W, Schreuer M, Waser M, Maisch S (2001). Exposure to farming in early life and development of asthma and allergy: a cross-sectional survey. Lancet (London, England).

[130] Ege MJ, Mayer M, Normand AC, Genuneit J, Cookson WOCM, Braun-Fahrländer C (2011). Exposure to environmental microorganisms and childhood asthma. N Engl J Med.

[131] von Mutius E, Vercelli D (2010). Farm living: effects on childhood asthma and allergy. Nat Rev Immunol.

[132] von Mutius E (2016). The microbial environment and its influence on asthma prevention in early life. J Allergy Clin Immunol.

[133] Perkin MR, Strachan DP (2006). Which aspects of the farming lifestyle explain the inverse association with childhood allergy?. J Allergy Clin Immunol.

[134] Dick S, Friend A, Dynes K, AlKandari F, Doust E, Cowie H (2014). A systematic review of associations between environmental exposures and development of asthma in children aged up to 9 years. BMJ Open.

[135] O’Connor GT, Lynch SV, Bloomberg GR, Kattan M, Wood RA, Gergen PJ (2018). Early-life home environment and risk of asthma among inner-city children. J Allergy Clin Immunol.

[136] Roduit C, Wohlgensinger J, Frei R, Bitter S, Bieli C, Loeliger S (2011). Prenatal animal contact and gene expression of innate immunity receptors at birth are associated with atopic dermatitis. J Allergy Clin Immunol.

[137] Ege MJ, Bieli C, Frei R, van Strien RT, Riedler J, Ublagger E (2006). Prenatal farm exposure is related to the expression of receptors of the innate immunity and to atopic sensitization in school-age children. J Allergy Clin Immunol.

[138] Loss G, Bitter S, Wohlgensinger J, Frei R, Roduit C, Genuneit J (2012). Prenatal and early-life exposures alter expression of innate immunity genes: The PASTURE cohort study. J Allergy Clin Immunol.

[139] Ege MJ, Herzum I, Büchele G, Krauss-Etschmann S, Lauener RP, Roponen M (2008). Prenatal exposure to a farm environment modifies atopic sensitization at birth. J Allergy Clin Immunol.

[140] Ege MJ, Mayer M, Schwaiger K, Mattes J, Pershagen G, van Hage M (2012). Environmental bacteria and childhood asthma. Allergy.

[141] Hagner S, Harb H, Zhao M, Stein K, Holst O, Ege MJ (2013). Farm-derived G ram-positive bacterium S taphylococcus sciuri W 620 prevents asthma phenotype in HDM-and OVA-exposed mice. Allergy.

[142] Kirjavainen PV, Karvonen AM, Adams RI, Täubel M, Roponen M, Tuoresmäki P (2019). Farm-like indoor microbiota in non-farm homes protects children from asthma development. Nat Med.

[143] Müller-Rompa SEK, Markevych I, Hose AJ, Loss G, Wouters IM, Genuneit J (2018). An approach to the asthma-protective farm effect by geocoding: Good farms and better farms. Pediatr Allergy Immunol.

[144] Brick T, Schober Y, Böcking C, Pekkanen J, Genuneit J, Loss G (2016). ω-3 fatty acids contribute to the asthma-protective effect of unprocessed cow’s milk. J Allergy Clin Immunol.

[145] Braun-Fahrländer C, Riedler J, Herz U, Eder W, Waser M, Grize L (2002). Environmental exposure to endotoxin and its relation to asthma in school-age children. N Engl J Med.

[146] Peters M, Kauth M, Schwarze J, Körner-Rettberg C, Riedler J, Nowak D (2006). Inhalation of stable dust extract prevents allergen induced airway inflammation and hyperresponsiveness. Thorax.

[147] Ege MJ, Frei R, Bieli C, Schram-Bijkerk D, Waser M, Benz MR (2007). Not all farming environments protect against the development of asthma and wheeze in children. J Allergy Clin Immunol.

[148] Stein MM, Hrusch CL, Gozdz J, Igartua C, Pivniouk V, Murray SE (2016). Innate immunity and asthma risk in Amish and Hutterite farm children. N Engl J Med.

[149] Reuter S, Dehzad N, Martin H, Bohm L, Becker M, Buhl R (2012). TLR3 but not TLR7/8 ligand induces allergic sensitization to inhaled allergen. J Immunol.

[150] Nigo YI, Yamashita M, Hirahara K, Shinnakasu R, Inami M, Kimura M (2006). Regulation of allergic airway inflammation through Toll-like receptor 4-mediated modification of mast cell function. Proc Natl Acad Sci USA.

[151] Thorne PS (2021). Environmental endotoxin exposure and asthma. J Allergy Clin Immunol.

[152] Thorne PS, Mendy A, Metwali N, Salo P, Co C, Jaramillo R (2015). Endotoxin exposure: predictors and prevalence of associated asthma outcomes in the United States. Am J Respir Crit Care Med.

[153] Mendy A, Wilkerson J, Salo PM, Weir CH, Feinstein L, Zeldin DC (2019). Synergistic association of house endotoxin exposure and ambient air pollution with asthma outcomes. Am J Respir Crit Care Med.

[154] Eldridge MW, Peden DB (2000). Allergen provocation augments endotoxin-induced nasal inflammation in subjects with atopic asthma. J Allergy Clin Immunol.

[155] Schaumann F, Müller M, Braun A, Luettig B, Peden DB, Hohlfeld JM (2008). Endotoxin augments myeloid dendritic cell influx into the airways in patients with allergic asthma. Am J Respir Crit Care Med.

[156] Hammad H, Lambrecht BN (2008). Dendritic cells and epithelial cells: linking innate and adaptive immunity in asthma. Nat Rev Immunol.

[157] Schuijs MJ, Willart MA, Vergote K, Gras D, Deswarte K, Ege MJ (2015). Farm dust and endotoxin protect against allergy through A20 induction in lung epithelial cells. Science.

[158] Krusche J, Twardziok M, Rehbach K, Böck A, Tsang MS, Schröder PC (2019). TNF-α–induced protein 3 is a key player in childhood asthma development and environment-mediated protection. J Allergy Clin Immunol.

[159] van der Vlugt LEPM, Eger K, Müller C, Ninaber DK, Zarcone MC, Amatngalim GD (2018). Farm dust reduces viral load in human bronchial epithelial cells by increasing barrier function and antiviral responses. J Allergy Clin Immunol.

[160] Frei R, Ferstl R, Roduit C, Ziegler M, Schiavi E, Barcik W (2018). Exposure to nonmicrobial N-glycolylneuraminic acid protects farmers’ children against airway inflammation and colitis. J Allergy Clin Immunol.

[161] Peters M, Kauth M, Scherner O, Gehlhar K, Steffen I, Wentker P (2010). Arabinogalactan isolated from cowshed dust extract protects mice from allergic airway inflammation and sensitization. J Allergy Clin Immunol.

[162] Roth-Walter F, Afify SM, Pacios LF, Blokhuis BR, Redegeld F, Regner A (2021). Cow’s milk protein β-lactoglobulin confers resilience against allergy by targeting complexed iron into immune cells. J Allergy Clin Immunol.

[163] Blaser MJ, Falkow S (2009). What are the consequences of the disappearing human microbiota?. Nat Rev Microbiol.

[164] Linz B, Balloux F, Moodley Y, Manica A, Liu H, Roumagnac P (2007). An African origin for the intimate association between humans and Helicobacter pylori. Nature.

[165] Bik EM, Eckburg PB, Gill SR, Nelson KE, Purdom EA, Francois F (2006). Molecular analysis of the bacterial microbiota in the human stomach. Proc Natl Acad Sci U S A.

[166] Suerbaum S, Michetti P (2002). Helicobacter pylori infection. N Engl J Med.

[167] Hooi JKY, Lai WY, Ng WK, Suen MMY, Underwood FE, Tanyingoh D (2017). Global prevalence of Helicobacter pylori infection: systematic review and meta-analysis. Gastroenterology.

[168] Sachs G, Weeks DL, Melchers K, Scott DR (2003). The gastric biology of Helicobacter pylori. Annu Rev Physiol.

[169] Eaton KA, Morgan DR, Krakowka S (1992). Motility as a factor in the colonisation of gnotobiotic piglets by Helicobacter pylori. J Med Microbiol.

[170] Bollmann R, Seeburg A, Parschau J, Schönian G, Sokolowska-Köhler W, Halle E (1997). Genotypic and phenotypic determination of five virulence markers in clinical isolates of Escherichia coli. FEMS Immunol Med Microbiol.

[171] Worku ML, Karim QN, Spencer J, Sidebotham RL (2004). Chemotactic response of Helicobacter pylori to human plasma and bile. J Med Microbiol.

[172] Bäckhed F, Rokbi B, Torstensson E, Zhao Y, Nilsson C, Seguin D (2003). Gastric mucosal recognition of Helicobacter pylori is independent of toll-like receptor 4. J Infect Dis.

[173] Lee SK, Stack A, Katzowitsch E, Aizawa SI, Suerbaum S, Josenhans C (2003). Helicobacter pylori flagellins have very low intrinsic activity to stimulate human gastric epithelial cells via TLR5. Microbes Infect.

[174] Bagheri B, Zambelli P, Vigentini I, Bauer FF, Setati ME (2018). Investigating the effect of selected non-saccharomyces species on wine ecosystem function and major volatiles. Front Bioeng Biotechnol..

[175] Chabaud M, Fossiez F, Taupin JL, Miossec P (1998). Enhancing effect of IL-17 on IL-1-induced IL-6 and leukemia inhibitory factor production by rheumatoid arthritis synoviocytes and its regulation by Th2 cytokines. J Immunol.

[176] Yamaoka Y (2010). Mechanisms of disease: Helicobacter pylori virulence factors. Nat Rev Gastroenterol Hepatol.

[177] Wroblewski LE, Peek RM, Wilson KT (2010). Helicobacter pylori and gastric cancer: factors that modulate disease risk. Clin Microbiol Rev.

[178] Lundgren A, Strömberg E, Sjöling A, Lindholm C, Enarsson K, Edebo A (2005). Mucosal FOXP3-expressing CD4+ CD25high regulatory T cells in Helicobacter pylori-infected patients. Infect Immun.

[179] Kandulski A, Wex T, Kuester D, Peitz U, Gebert I, Roessner A (2008). Naturally occurring regulatory T cells (CD4+, CD25high, FOXP3+) in the antrum and cardia are associated with higher H. pylori colonization and increased gene expression of TGF-β1. Helicobacter.

[180] Rad R, Brenner L, Bauer S, Schwendy S, Layland L, da Costa CP (2006). CD25+/Foxp3+ T cells regulate gastric inflammation and Helicobacter pylori colonization in vivo. Gastroenterology.

[181] Owyang SY, Zhang M, El-Zaatari M, Eaton KA, Bishu S, Hou G (2020). Dendritic cell-derived TGF-β mediates the induction of mucosal regulatory T-cell response to Helicobacter infection essential for maintenance of immune tolerance in mice. Helicobacter.

[182] Laur AM, Floch P, Chambonnier L, Benejat L, Korolik V, Giese A (2016). Regulatory T cells may participate in Helicobacter pylori persistence in gastric MALT lymphoma: lessons from an animal model. Oncotarget.

[183] Amedei A, Cappon A, Codolo G, Cabrelle A, Polenghi A, Benagiano M (2006). The neutrophil-activating protein of Helicobacter pylori promotes Th1 immune responses. J Clin Invest.

[184] Rimbara E, Mori S, Kim H, Shibayama K (2013). Role of γ-glutamyltranspeptidase in the pathogenesis of Helicobacter pylori infection. Microbiol Immunol.

[185] Abadi ATB (2017). Strategies used by helicobacter pylori to establish persistent infection. World J Gastroenterol.

[186] Sewald X, Gebert-Vogl B, Prassl S, Barwig I, Weiss E, Fabbri M (2008). Integrin subunit CD18 Is the T-lymphocyte receptor for the Helicobacter pylori vacuolating cytotoxin. Cell Host Microbe.

[187] Lina TT, Alzahrani S, Gonzalez J, Pinchuk IV, Beswick EJ, Reyes VE (2014). Immune evasion strategies used by Helicobacter pylori. World J Gastroenterol WJG.

[188] Oertli M, Noben M, Engler DB, Semper RP, Reuter S, Maxeiner J (2013). Helicobacter pylori γ-glutamyl transpeptidase and vacuolating cytotoxin promote gastric persistence and immune tolerance. Proc Natl Acad Sci U S A.

[189] Chen Y, Blaser MJ (2008). Helicobacter pylori colonization is inversely associated with childhood asthma. J Infect Dis.

[190] Chen Y, Blaser MJ (2007). Inverse associations of Helicobacter pylori with asthma and allergy. Arch Intern Med.

[191] Melby KK, Carlsen KL, Håland G, Samdal HH, Carlsen KH (2020). Helicobacter pylori in early childhood and asthma in adolescence. BMC Res Notes.

[192] Tsigalou C, Konstantinidis TG, Cassimos D, Karvelas A, Grapsa A, Tsalkidis A (2019). Inverse association between Helicobacter pylori infection and childhood asthma in Greece: a case-control study. Germs.

[193] Chen Y, Zhan X, Wang D (2021). Association between Helicobacter pylori and risk of childhood asthma: a meta-analysis of 18 observational studies. J Asthma.

[194] Wang Q, Yu C, Sun Y (2013). The association between asthma and Helicobacter pylori: a meta-analysis. Helicobacter.

[195] Zhou X, Wu J, Zhang G (2013). Association between Helicobacter pylori and asthma: a meta-analysis. Eur J Gastroenterol Hepatol.

[196] Chen C, Xun P, Tsinovoi C, He K (2017). Accumulated evidence on Helicobacter pylori infection and the risk of asthma: a meta-analysis. Ann Allergy, Asthma Immunol.

[197] Lim JH, Kim N, Lim SH, Kwon JW, Shin CM, Chang YS (2016). Inverse relationship between Helicobacter pylori infection and asthma among adults younger than 40 years: a cross-sectional study. Medicine (Baltimore).

[198] Taye B, Enquselassie F, Tsegaye A, Amberbir A, Medhin G, Fogarty A (2017). Association between infection with Helicobacter pylori and atopy in young Ethiopian children: a longitudinal study. Clin Exp Allergy.

[199] Reibman J, Marmor M, Filner J, Fernandez-Beros ME, Rogers L, Perez-Perez GI (2008). Asthma is inversely associated with Helicobacter pylori status in an urban population. PLoS ONE.

[200] den Hollander WJ, Sonnenschein-van der Voort AMM, Holster IL, de Jongste JC, Jaddoe VW, Hofman A (2016). Helicobacter pylori in children with asthmatic conditions at school age, and their mothers. Aliment Pharmacol Ther.

[201] Wang Y, Bi Y, Zhang L, Wang C (2012). Is Helicobacter pylori infection associated with asthma risk? A meta-analysis based on 770 cases and 785 controls. Int J Med Sci.

[202] Holster IL, Vila AMJ, Caudri D, den Hoed CM, Perez-Perez GI, Blaser MJ (2012). The impact of helicobacter pylori on atopic disorders in childhood. Helicobacter.

[203] Molina-Infante J, Gutierrez-Junquera C, Savarino E, Penagini R, Modolell I, Bartolo O (2018). Helicobacter pylori infection does not protect against eosinophilic esophagitis: results from a large multicenter case-control study. Am J Gastroenterol.

[204] Wang YC, Lin TY, Shang ST, Chen HJ, Kao CH, Wu CC (2017). Helicobacter pylori infection increases the risk of adult-onset asthma: a nationwide cohort study. Eur J Clin Microbiol Infect Dis.

[205] Fullerton D, Britton JR, Lewis SA, Pavord ID, McKeever TM, Fogarty AW (2009). Helicobacter pylori and lung function, asthma, atopy and allergic disease—a population-based cross-sectional study in adults. Int J Epidemiol.

[206] Arnold IC, Dehzad N, Reuter S, Martin H, Becher B, Taube C (2011). Helicobacter pylori infection prevents allergic asthma in mouse models through the induction of regulatory T cells. J Clin Invest.

[207] Oertli M, Sundquist M, Hitzler I, Engler DB, Arnold IC, Reuter S (2012). DC-derived IL-18 drives Treg differentiation, murine Helicobacter pylori-specific immune tolerance, and asthma protection. J Clin Invest.

[208] Engler DB, Reuter S, van Wijck Y, Urban S, Kyburz A, Maxeiner J (2014). Effective treatment of allergic airway inflammation with Helicobacter pylori immunomodulators requires BATF3-dependent dendritic cells and IL-10. Proc Natl Acad Sci.

[209] Kyburz A, Fallegger A, Zhang X, Altobelli A, Artola-Boran M, Borbet T (2018). Transmaternal Helicobacter pylori exposure reduces allergic airway inflammation in offspring through regulatory T cells.. J Allergy Clin Immunol.

[210] van Wijck Y, de Kleijn S, John-Schuster G, Mertens TCJ, Hiemstra PS, Müller A (2018). Therapeutic application of an extract of *Helicobacter pylori* ameliorates the development of allergic airway disease. J Immunol.

[211] Van Wijck Y, John-Schuster G, Van Schadewijk A, Van Den Oever RL, Obieglo K, Hiemstra PS (2019). Extract of Helicobacter pylori ameliorates parameters of airway inflammation and goblet cell hyperplasia following repeated allergen exposure. Int Arch Allergy Immunol.

[212] Reuter S, Raspe J, Uebner H, Contoyannis A, Pastille E, Westendorf AM (2023). Treatment with Helicobacter pylori-derived VacA attenuates allergic airway disease. Front Immunol.

[213] Raspe J, Schmitz MS, Barbet K, Caso GC, Cover TL, Müller A (2023). Therapeutic properties of Helicobacter pylori-derived vacuolating cytotoxin A in an animal model of chronic allergic airway disease. Respir Res.

[214] Altobelli A, Bauer M, Velez K, Cover TL, Müller A (2019). Helicobacter pylori VacA targets myeloid cells in the gastric lamina propria to promote peripherally induced regulatory T-cell differentiation and persistent infection. MBio.

[215] Zhou S, Huang Y, Liang B, Dong H, Yao S, Chen Y (2017). Systemic and mucosal pre-administration of recombinant Helicobacter pylori neutrophil-activating protein prevents ovalbumin-induced allergic asthma in mice. FEMS Microbiol Lett.

[216] Harhay MO, Horton J, Olliaro PL (2010). Epidemiology and control of human gastrointestinal parasites in children. Expert Rev Anti Infect Ther.

[217] Bohnacker S, Troisi F, de ReyesJiménez M, Esser-von Bieren J (2020). What can parasites tell us about the pathogenesis and treatment of asthma and allergic diseases. Front Immunol.

[218] Smits HH, Everts B, Hartgers FC, Yazdanbakhsh M (2010). Chronic helminth infections protect against allergic diseases by active regulatory processes. Curr Allergy Asthma Rep.

[219] van den Biggelaar AHJ, van Ree R, Rodrigues LC, Lell B, Deelder AM, Kremsner PG (2000). Decreased atopy in children infected with Schistosoma haematobium: a role for parasite-induced interleukin-10. Lancet.

[220] Araujo MI, Lopes AA, Medeiros M, Cruz ÁA, Sousa-Atta L, Solé D (2000). Inverse association between skin response to aeroallergens and Schistosoma mansoni infection. Int Arch Allergy Immunol.

[221] Cooper PJ, Chico ME, Rodrigues LC, Ordonez M, Strachan D, Griffin GE (2003). Reduced risk of atopy among school-age children infected with geohelminth parasites in a rural area of the tropics. J Allergy Clin Immunol.

[222] Rujeni N, Nausch N, Bourke CD, Midzi N, Mduluza T, Taylor DW (2012). atopy is inversely related to schistosome infection intensity: a comparative study in Zimbabwean villages with distinct levels of Schistosoma haematobium infection. Int Arch Allergy Immunol.

[223] Cooper PJ, Chico ME, Vaca MG, Sandoval CA, Loor S, Amorim LD (2017). Effect of early-life geohelminth infections on the development of wheezing at 5 years of age. Am J Respir Crit Care Med.

[224] Medeiros MJ, Figueiredo JP, Almeida MC, Matos MA, Araújo MI, Cruz AA (2003). Schistosoma mansoni infection is associated with a reduced course of asthma. J Allergy Clin Immunol.

[225] Li S, Jin X, Yan C, Wu S, Jiang F, Shen X (2010). Habitual snoring in school-aged children: environmental and biological predictors. Respir Res.

[226] Takeuchi H, Zaman K, Takahashi J, Yunus M, Chowdhury HR, El AS (2008). High titre of anti-Ascaris immunoglobulin E associated with bronchial asthma symptoms in 5-year-old rural Bangladeshi children. Clin Exp Allergy.

[227] Takeuchi H, Khan AF, Yunus M, Hasan MI, Hawlader MDH, Takanashi S (2016). Anti-Ascaris immunoglobulin E associated with bronchial hyper-reactivity in 9-year-old rural Bangladeshi children. Allergol Int.

[228] McKay DM (2015). Not all parasites are protective. Parasite Immunol.

[229] Smits HH, Hammad H, van Nimwegen M, Soullie T, Willart MA, Lievers E (2007). Protective effect of Schistosoma mansoni infection on allergic airway inflammation depends on the intensity and chronicity of infection. J Allergy Clin Immunol.

[230] Favoretto BC, Casabuono AAC, Portes-Junior JA, Jacysyn JF, Couto AS, Faquim-Mauro EL (2017). High molecular weight components containing N-linked oligosaccharides of Ascaris suum extract inhibit the dendritic cells activation through DC-SIGN and MR. Mol Immunol.

[231] Hawlader MDH, Ma E, Noguchi E, Itoh M, Arifeen SE, Persson LÅ (2014). Ascaris lumbricoids infection as a risk factor for asthma and atopy in rural Bangladeshi children. Trop Med Health.

[232] Hunninghake GM, Soto-Quiros ME, Avila L, Ly NP, Liang C, Sylvia JS (2007). Sensitization to Ascaris lumbricoides and severity of childhood asthma in Costa Rica. J Allergy Clin Immunol.

[233] Palmer LJ, Celedón JC, Weiss ST, Wang B, Fang Z, Xu X (2002). Ascaris lumbricoides infection is associated with increased risk of childhood asthma and atopy in rural China. Am J Respir Crit Care Med.

[234] Aghaei S, Riahi SM, Rostami A, Mohammadzadeh I, Javanian M, Tohidi E (2018). Toxocara spp. infection and risk of childhood asthma: a systematic review and meta-analysis. Acta Trop.

[235] Evans H, Mitre E (2015). Worms as therapeutic agents for allergy and asthma: understanding why benefits in animal studies have not translated into clinical success. J Allergy Clin Immunol.

[236] Mangan NE, van Rooijen N, McKenzie ANJ, Fallon PG (2006). Helminth-modified pulmonary immune response protects mice from allergen-induced airway hyperresponsiveness. J Immunol.

[237] Mo HM, Lei JH, Jiang ZW, Wang CZ, Cheng YL, Li YL (2008). Schistosoma japonicum infection modulates the development of allergen-induced airway inflammation in mice. Parasitol Res.

[238] Liu P, Li J, Yang X, Shen Y, Zhu Y, Wang S (2010). Helminth infection inhibits airway allergic reaction and dendritic cells are involved in the modulation process. Parasite Immunol.

[239] Liu JY, Li LJY, Yang XZ, Li J, Zhong G, Wang J (2011). Adoptive transfer of dendritic cells isolated from helminth-infected mice enhanced T regulatory cell responses in airway allergic inflammation. Parasite Immunol.

[240] van der Vlugt LEPM, Labuda LA, Ozir-Fazalalikhan A, Lievers E, Gloudemans AK, Liu KY (2012). Schistosomes induce regulatory features in human and mouse CD1dhi B cells: inhibition of allergic inflammation by IL-10 and regulatory T cells. PLoS ONE.

[241] Layland LE, Straubinger K, Ritter M, Loffredo-Verde E, Garn H, Sparwasser T (2013). Schistosoma mansoni-mediated suppression of allergic airway inflammation requires patency and Foxp3+ Treg cells. PLoS Negl Trop Dis.

[242] Haeberlein S, Obieglo K, Ozir-Fazalalikhan A, Chayé MAM, Veninga H, van der Vlugt LEPM (2017). Schistosome egg antigens, including the glycoprotein IPSE/alpha-1, trigger the development of regulatory B cells. PLoS Pathog.

[243] Obieglo K, Schuijs MJ, Ozir-Fazalalikhan A, Otto F, van Wijck Y, Boon L (2018). Isolated Schistosoma mansoni eggs prevent allergic airway inflammation. Parasite Immunol.

[244] Joyce KL, Morgan W, Greenberg R, Nair MG (2012). Using eggs from Schistosoma mansoni as an in vivo model of helminth-induced lung inflammation. JoVE J Vis Exp..

[245] Yang J, Zhao J, Yang Y, Zhang L, Yang X, Zhu X (2007). Schistosoma japonicum egg antigens stimulate CD4+ CD25+ T cells and modulate airway inflammation in a murine model of asthma. Immunology.

[246] Zaccone P, Burton O, Miller N, Jones FM, Dunne DW, Cooke A (2009). Schistosoma mansoni egg antigens induce Treg that participate in diabetes prevention in NOD mice. Eur J Immunol.

[247] Zaccone P, Fehérvári Z, Jones FM, Sidobre S, Kronenberg M, Dunne DW (2003). Schistosoma mansoni antigens modulate the activity of the innate immune response and prevent onset of type 1 diabetes. Eur J Immunol.

[248] Zaccone P, Burton OT, Gibbs S, Miller N, Jones FM, Dunne DW (2010). Immune modulation by Schistosoma mansoni antigens in NOD mice: effects on both innate and adaptive immune systems. J Biomed Biotechnol.

[249] Marinho FV, Alves CC, de Souza SC, da Silva CMG, Cassali GD, Oliveira SC (2016). Schistosoma mansoni Tegument (Smteg) induces IL-10 and modulates experimental airway inflammation. PLoS ONE.

[250] Ren J, Hu L, Yang J, Yang L, Gao F, Lu P (2016). Novel T-cell epitopes on Schistosoma japonicum SjP40 protein and their preventive effect on allergic asthma in mice. Eur J Immunol.

[251] Zhang W, Li L, Zheng Y, Xue F, Yu M, Ma Y (2019). Schistosoma japonicum peptide SJMHE1 suppresses airway inflammation of allergic asthma in mice. J Cell Mol Med.

[252] Gao X, Ren X, Wang Q, Yang Z, Li Y, Su Z (2019). Critical roles of regulatory B and T cells in helminth parasite-induced protection against allergic airway inflammation. Clin Exp Immunol.

[253] Schnoeller C, Rausch S, Pillai S, Avagyan A, Wittig BM, Loddenkemper C (2008). A helminth immunomodulator reduces allergic and inflammatory responses by induction of IL-10-producing macrophages. J Immunol.

[254] Ryan SM, Eichenberger RM, Ruscher R, Giacomin PR, Loukas A (2020). Harnessing helminth-driven immunoregulation in the search for novel therapeutic modalities. PLOS Pathog.

